# Chronobiology of neurotropic viruses: rhythmic viral entry and arrhythmic host clocks

**DOI:** 10.1038/s41421-026-00867-8

**Published:** 2026-02-10

**Authors:** Shaowei Zeng, Qian Zhang, Xue Yang, Linyue Lv, Yuelan Zhang, Zhuyou Zhang, Qinyang Wang, Min-Hua Luo, Martin Dorf, Shitao Li, Ling Zhao, Bishi Fu

**Affiliations:** 1 Department of Rheumatology and Immunology, State Key Laboratory of Virology and Biosafety, Zhongnan Hospital, Wuhan University, Wuhan, Hubei China; 2 Frontier Science Center for Immunology and Metabolism, Medical Research Institute, School of Medicine, Wuhan University, Wuhan, Hubei China; 3 Institute of Medical Biology, Chinese Academy of Medical Sciences and Peking Union Medical College, Kunming, Yunnan China; 4 School of Life Sciences, Yunnan University, Kunming, Yunnan China; 5 State Key Laboratory of Virology and Biosafety, Wuhan Institute of Virology, Chinese Academy of Sciences, Wuhan, Hubei China; 6 Department of Microbiology & Immunobiology, Harvard Medical School, Boston, MA USA; 7 Department of Microbiology and Immunology, Tulane University, New Orleans, LA USA; 8 State Key Laboratory of Agricultural Microbiology, Huazhong Agricultural University, Wuhan, Hubei China; 9 Key Laboratory of Preventive Veterinary Medicine of Hubei Province, Huazhong Agricultural University, Wuhan, Hubei China

**Keywords:** Circadian rhythms, Mechanisms of disease, Ubiquitylation

## Abstract

Neurotropic viruses invade neural tissues, resulting in severe diseases such as poliomyelitis, rabies, herpesviral encephalitis, and viral meningitis. Given this neurotropism, we investigated whether the infection of the host by these viruses is under circadian control. In this study, we found that the expression of most neurotropic virus receptors exhibits rhythmicity across cells, cerebral organoids, and animal models, with host cell susceptibility modulated by the circadian clock. We identified E2F8 as a clock-controlled gene that mediates the indirect regulation of the circadian clock on neurotropic viruses. Notably, E2F8 regulated the expression of core clock components by binding directly to the promoters of *REV-ERBα* and *PER2*, suggesting its role as a potential modulator of circadian rhythms. Additionally, we revealed a seldom-recognized viral strategy to accelerate viral replication in the host: rabies virus disrupts the host circadian clock system primarily through its glycoprotein hijacking the E3 ubiquitin ligase HUWE1 to inhibit proteasomal degradation of REV-ERBα. These findings increase our understanding of the interactions between circadian systems and neurotropic viral dynamics and highlight the potential of chronotherapy for improved antiviral treatments.

## Introduction

Viruses with neurotropic or neuroinvasive potential are pathogens capable of invading the central and peripheral nervous systems, precipitating severe neurological disorders^1,2^. These infections impose substantial disease and economic burdens globally, posing significant challenges to health care systems because of morbidity and mortality^3–6^. For instance, the rabies virus (RABV) causes approximately 59,000 deaths annually worldwide, with an estimated $8.6 billion spent annually on the global response^7,8^. Human cytomegalovirus (HCMV) infections, ranging from self-limiting to fatal, are a major public health concern^9–11^. Recognition and interaction between viruses and receptors are critical initial steps in the viral life cycle^12–16^. Viruses with neurotropic or neuroinvasive potential utilize a variety of receptors to enter and infect nerve cells, and effective inhibition of virus‒host cell interactions can prevent viral entry, thereby alleviating or eradicating the infection^17–19^. Monoclonal antibody drugs formulated on this basis have succeeded but encounter multiple challenges, including the risk of allergic reactions, high production costs, and potential drug resistance^20,21^. Consequently, exploring the host’s intrinsic physiological mechanisms that can modulate viral receptors is crucial for the development of new antiviral strategies.

Mammalian circadian clocks are biological systems that regulate the daily rhythms of physiology and behavior in animals^22–24^; these clocks include a central clock in the brain and peripheral clocks in various tissues and organs^25–27^. Synchronized by external cues, such as light^28,29^, food^30,31^, and temperature^32^, the signaling pathway of the mammalian circadian clock, which is present in nearly all cells, is governed primarily by transcription-translation feedback loops (TTFLs). The transcription activators BMAL1 and CLOCK trigger the expression of numerous transcripts, including their inhibitors, such as REV-ERBα/β, PER, and CRY, which repress BMAL1 and are vital for maintaining a functional clock^33,34^. Growing evidence suggests that circadian pathway components may regulate viral entry and the viral gene replication process. Additionally, the circadian clock is instrumental in recognizing and eliminating viral pathogens through the modulation of immune system components, such as cytokines, interferons, natural killer cells, and T cells^35,36^. Furthermore, the circadian clock affects the efficacy of antiviral drugs and vaccines through its influence on their pharmacokinetics^37,38^. The nervous system plays a pivotal role in maintaining the homeostasis of an organism’s circadian rhythms^39^, and the life cycles of neurotropic viruses are inextricably linked to the nervous system^40^. In addition, as host cellular proteins, viral receptors are inevitably regulated by host physiological processes^41,42^. In mammals, approximately 50% of genes are controlled by the circadian clock^43–45^. Consequently, we propose that circadian regulation of receptors of viruses with neurotropic or neuroinvasive potential is a widespread phenomenon. However, there is a lack of systematic research on the relationship between the circadian clock and receptors of viruses with neurotropic or neuroinvasive potential.

In this study, we revealed the dynamic nature of host susceptibility to viruses with neurotropic or neuroinvasive potential and its intricate association with the circadian clock. We used the cleavage under targets and tagmentation (CUT&Tag) technique to investigate the interaction between BMAL1 and DNA in cerebral organoids and detected distinct BMAL1 binding peaks in 32 receptor promoter regions of viruses with neurotropic or neuroinvasive potential. Moreover, we found that the expression of receptors for viruses with neurotropic or neuroinvasive potential and host susceptibility to these viruses exhibited circadian rhythmicity across species and were regulated by core clock transcriptional regulators, particularly BMAL1 and REV-ERBα. Additionally, we revealed that the cell cycle regulator E2F8 acted as an intermediary, mediating the rhythmic output of REV-ERBα to the RABV receptor p75NTR. Moreover, we demonstrated that E2F8 bound to the promoters of *REV-ERBα* and *PER2*, thereby leading to the dysregulation of the expression of multiple clock components. Our study is the first to report that E2F8 participates in viral infection of the host by directly repressing the transcription of receptor genes or by affecting circadian clock components. Furthermore, we discovered that the circadian rhythm of host infected with RABV was disrupted, with the main mechanism being that the RABV glycoprotein hijacks the E3 ubiquitin ligase HUWE1, thereby inhibiting the ubiquitin-proteasome-mediated degradation of REV-ERBα, which in turn disrupts BMAL1 homeostasis. Finally, by manipulating light exposure or using CRISPR-Cas9 technology, we constructed three mouse models related to the circadian clock and demonstrated that both the timing of viral infection and the integrity of a functional circadian system were critical determinants of infection outcome. Additionally, REV-ERBα emerged as a particularly promising target for limiting RABV infection. Ultimately, our findings deepen our understanding of potential neurotropic or neuroinvasive virus–host dynamics and may guide the development of novel prophylactic and therapeutic strategies.

## Results

### Neurotropic viral receptors exhibit circadian rhythmicity across different models

**Fig. 1:**
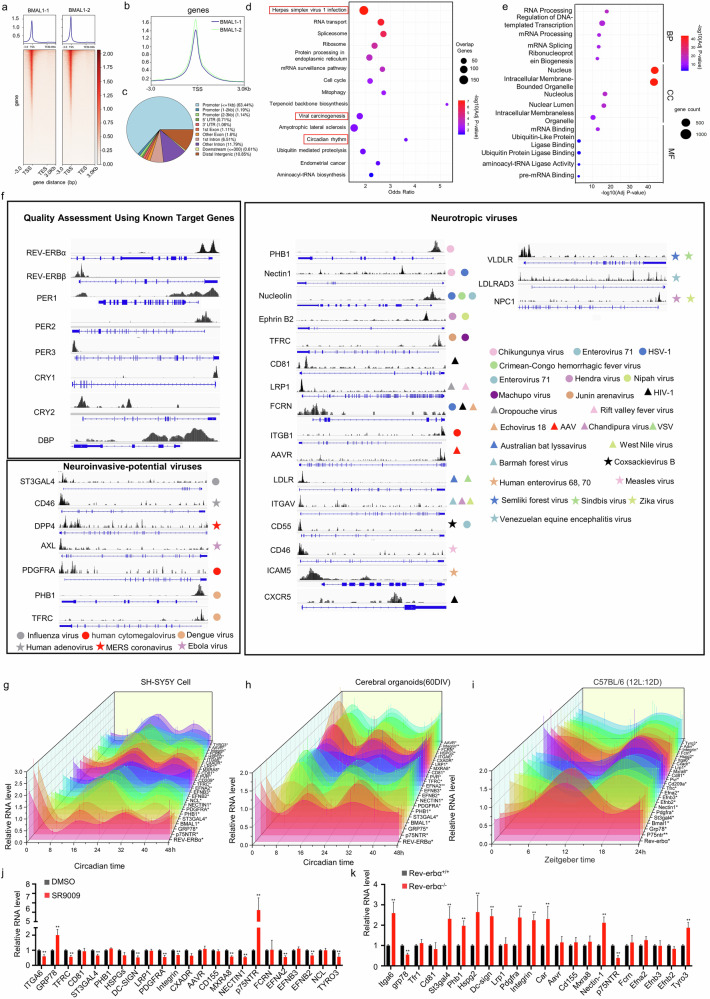
Neurotropic viral receptors exhibit circadian rhythmicity across different models. Cerebral organoids (60 days in vitro (DIV)) were synchronized with 100 nM hydrocortisone and collected at CT7 for CUT&Tag-seq assays to screen the binding sites of BMAL1. **a** Heatmaps depicting the distribution of BMAL1 peaks (replicates BMAL1-1 and BMAL1-2) centered on the TTS, spanning ‒3.0 kb to +3.0 kb, with peak intensity indicated by a color gradient. **b** Average density profiles of BMAL1 binding (BMAL1-1 in dark blue; BMAL1-2 in light blue) across all genes, showing peak enrichment near the TSS. **c** Pie chart illustrating the genomic distribution of BMAL1 peaks. **d**, **e** KEGG pathway enrichment analysis (**d**) or Gene Ontology enrichment analysis (**e**) of genes associated with BMAL1 in promoter binding peaks. **f** Integrative Genomics Viewer tracks of CUT&Tag data showing enriched BMAL1 occupancy at the promoters of known BMAL1 target genes (e.g., *REVERBα* and *PER1*) and at receptors associated with viruses with neurotropic or neuroinvasive potential. **g**, **h** SH-SY5Y cells were synchronized with 100 nM dexamethasone and sampled every 8 h starting from CT0 for 48 h (**g**). Cerebral organoids (60 DIV) were synchronized with 100 nM hydrocortisone and collected every 8 h starting from CT0 (**h**). Total RNA was extracted, and the mRNA levels of *BMAL1*, *REV-ERBα*, and selected neurotropic viral receptors were quantified by qRT-PCR. **i** C57BL/6J mice were housed under a 12 h:12 h light–dark cycle, and whole-brain tissue was harvested every 8 h starting from ZT0 over 24 h. mRNA levels of *Bmal1*, *Rev-erbα*, and neurotropic viral receptors were measured by qRT-PCR and normalized to the overall mean of each gene across the cycle. **j**, **k** mRNA levels of neurotropic virus receptors were measured by qRT-PCR using total RNA extracted from SH-SY5Y cells treated with 10 μM SR9009 for 24 h (**j**) or from the brain tissue of Rev-erbα knockout mice (**k**). All experiments were performed in triplicate. The data are normalized to the mean value within each group and are presented as the mean ± standard error of the mean (SEM). Statistical significance was assessed using independent-sample *t*-tests, whereas rhythmicity was evaluated by autoregressive spectral estimation (ARS) or cosine fitting combined with joint significance testing of harmonic components (CFJHC). *P < 0.05, **P < 0.01.

The nervous system is crucial for both neurotropic viral infection and the synchronization of the central clock with peripheral circadian clocks^1,2,39^. Although the circadian clock is known to regulate certain viral infections^46–51^, whether this regulatory role extends to all neurotropic viruses remains unclear. Given that viral receptors are critical for viral infection^12,14,15^, the promoters of well-characterized receptors for neurotropic viruses were examined (Supplementary Table S1). E-BOX or RORE motifs, which are key binding motifs for circadian transcription factors^33^, were present in the promoters of all the receptors (Supplementary Fig. S1a). BMAL1 is a core transcription factor that partners with the CLOCK protein to activate the expression of genes, and its genome-wide occupancy rhythms peak at CT6–CT8^33,34^. To investigate the direct regulation of neurotropic viral receptors by the circadian clock, cerebral organoids (Supplementary Fig. S1b) were synchronized with hydrocortisone^52^. The results demonstrated that treatment with 100 nM hydrocortisone effectively reset the circadian oscillations of the organoids, with *BMAL1* mRNA levels peaking around CT7 within the CT1–CT12 interval (Supplementary Fig. S1c). Accordingly, cerebral organoids were sampled at CT7 for a CUT&Tag assay using an anti-BMAL1 antibody, which revealed a noticeable enrichment in the transcription start site (TSS) region (Fig. 1a, b), with ~63% of BMAL1 peaks located in the promoter regions (Fig. 1c). Kyoto Encyclopedia of Genes and Genomes (KEGG) pathway analysis of genes associated with these promoter-associated peaks revealed significant enrichment for “Circadian Rhythm,” confirming the reliability of our CUT&Tag-seq data. Furthermore, pathways related to viral infection, including “Herpes Simplex Virus 1 (HSV-1) infection” and “Viral carcinogenesis”, were also significantly enriched (Fig. 1d). Gene Ontology (GO) analysis of peak-associated genes demonstrated enrichment for terms associated with RNA processing, nuclear components, and ligase activity (Fig. 1e). To specifically determine whether BMAL1 binds to the promoters of neurotropic virus receptors, we performed a detailed analysis of the identified peaks. First, we analyzed classical BMAL1 target genes (e.g., *REV-ERBα* and *PER1*) and observed pronounced BMAL1 binding peaks at these loci, further confirming that CT7 represents an appropriate circadian time point for investigating BMAL1–promoter interactions. We subsequently detected significant BMAL1 binding signals in the promoter regions of 23 receptors, including PDGFRA, ITGB1, and AXL. These receptors correspond to 32 different viruses with neurotropic or neuroinvasive potential, such as HCMV, Ebola virus (EBOV), and Chikungunya virus (Fig. 1f), indicating a potential regulatory function of the circadian clock on neurotropic viral receptors.

Additionally, we analyzed RNA-seq data from mouse dorsal hippocampus (GSE222744^53^) and salivary gland tissues (GSE234789 (not published)), as well as Calu-3 and U2OS cells (GSE176393^54^ and GSE248721^55^), from the NCBI Gene Expression Omnibus (GEO) database. The results revealed that the expression of most neurotropic virus receptors exhibited significant oscillations at different Zeitgeber times (Supplementary Fig. S1d). Furthermore, the expression of these receptors was either upregulated or downregulated following pharmacological activation of REV-ERBα with SR9009 or upon Rev-erbα knockout (KO) (Supplementary Fig. S1e), with the expression of the RABV receptor *p75NTR* showing the most substantial changes. To confirm the pervasive nature of this circadian regulation, we utilized three distinct experimental models: the human neuroblastoma cell line SH-SY5Y, human cerebral organoids, and C57BL/6 mice. Synchronization was achieved through a 100 nM dexamethasone pulse^56,57^, a 100 nM hydrocortisone pulse^52^, and a 12-h light/dark cycle^58^, respectively. In all three models, the mRNA levels of the investigated neurotropic virus receptors exhibited significant rhythmic expression (Fig. 1g–i). Using a Euclidean distance algorithm, we clustered the expression rhythms of these receptors into two distinct groups. One group, which included the HCMV receptors *ITGB1* and *PDGFRA*, oscillated in phase with the expression profile of *BMAL1*. The other group, which included the RABV receptor *p75NTR*, oscillated in phase with *REV-ERBα* (Supplementary Fig. S1f–h). We subsequently quantified the mRNA levels of these receptors in SH-SY5Y cells treated with DMSO or the REV-ERBα agonist SR9009, as well as in brain tissues from wild-type (WT) or Rev-erbα-KO mice. Under our experimental conditions, no significant adverse effects of SR9009 on SH-SY5Y cell viability were detected (Supplementary Fig. S1i). We found that pharmacological activation of REV-ERBα with SR9009 in SH-SY5Y cells or the brains of Rev-erbα-KO mice significantly altered the expression of most receptors. The most pronounced effect was again observed for the RABV receptor *p75NTR* (Fig. 1j, k). Taken together, these results demonstrate that the expression of neurotropic viral receptors is robustly regulated by the circadian clock in a conserved manner across different models and species.

### Host cell susceptibility to neurotropic viruses is regulated by the circadian clock

**Fig. 2:**
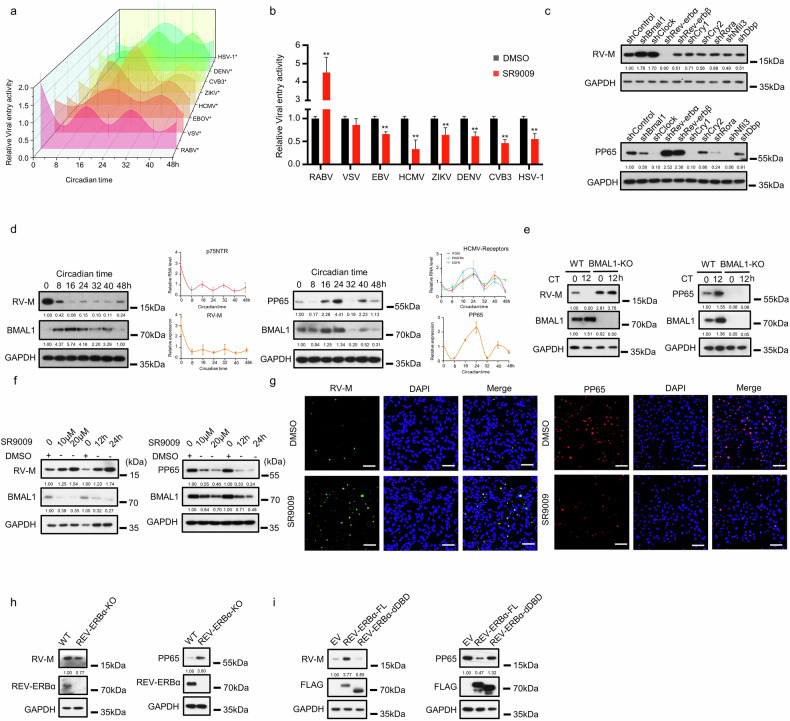
Susceptibility of host cells to neurotropic viruses is regulated by the circadian clock. **a**, **b** SH-SY5Y cells were infected with neurotropic viruses (RABV, VSV, ZIKV, or CVB3) or viruses with neuroinvasive potential (DENV). HEK293 cells were infected with viruses with neuroinvasive potential (EBOV) or neurotropic viruses (HSV-1). U-251 cells were infected with viruses with neuroinvasive potential (HCMV). The cells synchronized with 100 nM dexamethasone pulses were infected with the respective viruses at different circadian times (**a**), or the cells were infected with the illustrated virus after 24 h of treatment with 10 μm SR9009 (**b**). Among them, RABV, VSV, and EBOV were pseudoviruses, and their activity in invading host cells was characterized by detecting luciferase activity. The ability of HCMV, ZIKV, DENV, CVB3, and HSV-1 to invade host cells was characterized by detecting the abundance of viral proteins. **c** Lentiviral shRNA targeted circadian factors in SH-SY5Y/U-251 cells; after CVS-11/AD169 infection, RABV-M, HCMV-PP65, and GAPDH levels were measured by western blotting assay. **d** After 48 h of CVS-11/AD169 infection in synchronized SH-SY5Y/U-251 cells, protein expression was analyzed by western blotting assay. **e** CVS-11/AD169 infection in WT/BMAL1 KO SH-SY5Y/U-251 cells at CT0/CT12; RABV-M, BMAL1, GAPDH (SH-SY5Y), PP65, BMAL1, and GAPDH (U-251) expression was analyzed at 48 h by western blotting assay. **f** SR9009-treated SH-SY5Y/U-251 cells (10/20 μM, 24 h; 10 μM, 12/24 h) were infected with CVS-11/AD169; RABV-M, BMAL1, GAPDH (SH-SY5Y), PP65, BMAL1, and GAPDH (U-251) and quantified at 48 h by western blotting assay. **g** SR9009-treated SH-SY5Y/U-251 cells (10 μM, 24 h) infected with CVS-11/AD169, RABV-M or PP65, and DAPI were assessed by immunofluorescence staining. Scale bars, 50 μm. **h** CVS-11/AD169 infection in WT/REV-ERBα-KO SH-SY5Y/U-251 cells; RABV-M, PP65, REV-ERBα, and GAPDH levels were assessed at 48 h by western blotting assay. **i** CVS-11/AD169 infection in SH-SY5Y/U-251 cells expressing empty vector, REV-ERBα-FL, or REV-ERBα-dDBD; CVS-11 M, FLAG, GAPDH (SH-SY5Y), PP65, FLAG, and GAPDH (U-251) were measured at 48 h by western blotting assay. All experiments were performed in triplicate. The data are presented as the mean ± SEM. Statistical significance was determined using independent-sample *t*-tests, whereas rhythmicity was evaluated by ARS or CFJHC. *P < 0.05, **P < 0.01.

To investigate whether circadian clock regulation affects host susceptibility to viruses with neurotropic or neuroinvasive potential, we selected a panel of representative viruses for viral entry assays, including neurotropic viruses (RABV, vesicular stomatitis virus (VSV), Zika virus (ZIKV), Coxsackievirus B3 (CVB3), and HSV-1) and viruses with neuroinvasive potential (EBOV, dengue virus (DENV), and HCMV). As hypothesized, host cell susceptibility to these viruses exhibited a rhythmic pattern that mirrored the oscillatory expression of their respective receptors (Fig. 2a). We subsequently treated SH-SY5Y and U-251 cells with SR9009 to investigate the effect of REV-ERBα activation on viral entry into host cells; under our experimental conditions, SR9009 did not significantly decrease U-251 cell viability (Supplementary Fig. S2a). SR9009 treatment significantly increased RABV entry but inhibited the entry of the other viruses (Fig. 2b); these findings for ZIKV, DENV, and HSV-1 were consistent with those of previous reports^46,49^. For a more in-depth mechanistic study, we focused on the RNA virus RABV and the DNA virus HCMV, as their respective receptors exhibit expression patterns that align with the rhythms of the core clock components REV-ERBα and BMAL1. We used shRNA to knock down these clock genes in SH-SY5Y cells and the human glioblastoma multiforme cell line U-251 (Supplementary Fig. S2b). We subsequently infected the knockdown (KD) SH-SY5Y cells with the RABV CVS-11 strain and U-251 cells with the HCMV AD169 strain. Notably, REV-ERBα KD had the most potent effect, strongly inhibiting RABV infection while increasing HCMV infection (Fig. 2c). Within the circadian TTFL, RORA typically functions as a transcriptional activator, whereas REV-ERBα serves as a transcriptional repressor. Both proteins recognize and bind to identical or overlapping DNA motifs^33,34^. We observed that they exerted critical yet opposing effects on the outcome of HCMV infection in U-251 cells (Fig. 2c). In contrast, during the RABV infection of SH-SY5Y cells, only REV-ERBα knockdown resulted in a pronounced inhibitory effect (Fig. 2c). Subsequent experiments demonstrated that REV-ERBα overexpression or pharmacological activation with SR9009 markedly increased RABV infection in SH-SY5Y cells, whereas RORA overexpression or stimulation with SR1078 elicited only a modest inhibitory effect (Supplementary Fig. S2c).

For transcriptional activators within the TTFL, rhythmic binding to promoter regions of target genes enables the transmission of their oscillatory patterns to downstream gene expression. Interestingly, as shown in Fig. 1g–i, the rhythmic oscillation patterns of certain neurotropic viral receptors consistently aligned with those of *REV-ERBα* across different species. This raised the intriguing question of how REV-ERBα, which functions as a transcriptional repressor within the TTFL, could propagate its oscillatory rhythm to these neurotropic viral receptors. Consequently, our study focused primarily on elucidating the molecular mechanisms through which REV-ERBα governs the circadian regulation of neurotropic viral receptor rhythmic expression.

Furthermore, we observed that synchronizing U-251 cells with dexamethasone also induced rhythmic gene expression (Supplementary Fig. S2d). When synchronized SH-SY5Y and U-251 cells were infected with RABV or HCMV at different circadian times, we observed that the overall infection outcome was rhythmic (Fig. 2d). Deletion of BMAL1 is known to abolish cellular circadian rhythms^59,60^. Indeed, BMAL1 KO in SH-SY5Y and U-251 cells disrupted the rhythmic expression of REV-ERBα (Supplementary Fig. S2e) and, consequently, abolished the rhythmic susceptibility to RABV and HCMV, respectively (Fig. 2e).

Furthermore, SR9009 promoted RABV infection in SH-SY5Y cells and inhibited HCMV infection in U-251 cells in a dose- and time-dependent manner (Fig. 2f). We also noted that SR9009 treatment increased the number of RABV particles in infected SH-SY5Y cells but reduced the number of HCMV particles in infected U-251 cells (Fig. 2g). Conversely, RABV infection was markedly reduced in REV-ERBα-KO SH-SY5Y cells, whereas HCMV infection was significantly increased in REV-ERBα-KO U-251 cells (Fig. 2h). To determine whether the effect of REV-ERBα on viral infection depends on its transcriptional regulatory function, we engineered a mutant REV-ERBα lacking its DNA-binding domain (REV-ERBα-dDBD). Overexpression of full-length REV-ERBα (REV-ERBα-FL) in SH-SY5Y cells increased RABV infection, but this effect was absent in cells expressing the REV-ERBα-dDBD mutant. In contrast, overexpression of REV-ERBα-FL in U-251 cells suppressed HCMV infection, an effect that was also abrogated in cells expressing a mutant lacking the DNA-binding domain (Fig. 2i). Taken together, these data demonstrate that host cell susceptibility to neurotropic viruses is regulated by the circadian clock and that clock components play key divergent roles in RNA and DNA virus infection.

### E2F8 repression by REV-ERBα augments p75NTR expression and RABV entry

**Fig. 3:**
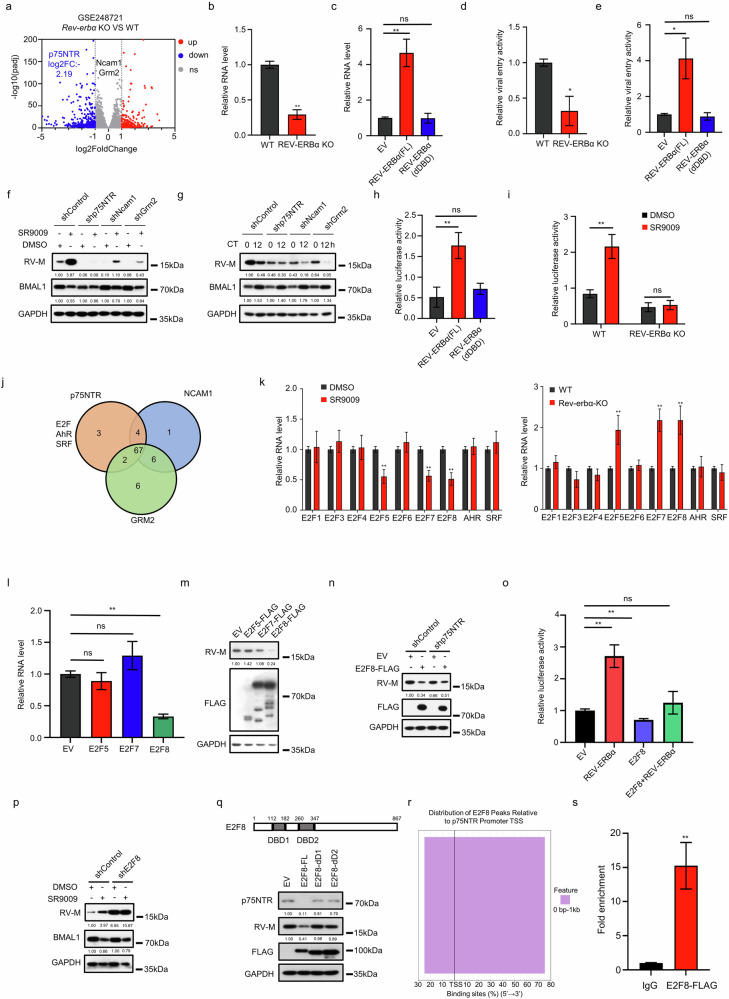
E2F8 repression by REV-ERBα augments p75NTR expression and RABV entry. **a** DEG analysis of RNA-seq data from U2OS WT/REV-ERBα-KO cell lines, as visualized via volcano plots. **b**, **c** qRT-PCR analysis of *p75NTR* mRNA levels in WT/REV-ERBα-KO SH-SY5Y cells (**b**) and SH-SY5Y cells stably overexpressing EV/REV-ERBα-FL/REV-ERBα-dDBD (**c**). *GAPDH* was used as the internal control. **d**, **e** RABV entry assay using the CVS-11 strain in WT/REV-ERBα-KO (**d**) and SH-SY5Y cells stably overexpressing EV/REV-ERBα-FL/REV-ERBα-dDBD (**e**). Viral entry was quantified by qRT-PCR measurement of RABV genome RNA. The detailed experimental procedures are described in the “Materials and Methods” section. **f**, **g** SH-SY5Y cells in which p75NTR, NCAM1, or GRM2 was knocked down or control cells were treated with DMSO/10 μM SR9009 for 24 h (**f**) or treated with a 100 nM dexamethasone pulse (**g**). Following infection with the RABV strain CVS-11 for 48 h, the protein levels of RABV-M, BMAL1, and GAPDH were analyzed by western blotting assay. **h** HEK293 cells were cotransfected with a luciferase reporter driven by the *p75NTR* promoter (‒2 kb) and plasmids encoding control, full-length REV-ERBα (REV-ERBα-FL), or REV-ERBα-dDBD. Luciferase activity was measured 48 h post-transfection. **i** WT and REV-ERBα-KO HEK293 cells were treated with DMSO or 10 μM SR9009 for 24 h and then transfected with the p75NTR promoter luciferase reporter. Luciferase activity was quantified 48 h later. **j** PROMO was used to predict transcription factor-binding sites at the *p75NTR*, *NCAM1*, and *GRM2* promoters. The results were visualized using a Venn diagram. **k** Total RNA was extracted from SH-SY5Y cells treated with DMSO or 10 μM SR9009 for 24 h or from WT and REV-ERBα-KO SH-SY5Y cells. qRT-PCR was performed to measure the mRNA levels of E2F family transcription factors, *AHR*, *SRF*, *BMAL1*, and *GAPDH*. **l** Total RNA was extracted from SH-SY5Y cells overexpressing empty vector (EV), E2F5, E2F7, or E2F8. The mRNA levels of *p75NTR* and *GAPDH* were measured by qRT-PCR. **m** SH-SY5Y cells overexpressing EV, E2F5, E2F7, or E2F8 were infected with CVS-11 for 48 h. The protein levels of RABV-M, FLAG-tagged proteins, and GAPDH were analyzed by western blotting assay. **n** SH-SY5Y cells overexpressing EV or E2F8 were infected with lentivirus for p75NTR knockdown or control. After 48 h of CVS-11 infection, the protein levels of RABV-M, FLAG-tagged proteins, and GAPDH were assessed by western blotting assay. **o** HEK293 cells were cotransfected with luciferase reporters driven by the *p75NTR* promoter and plasmids encoding EV, REV-ERBα, or E2F8. Luciferase activity was quantified 48 h post-transfection and normalized to that of the controls. **p** SH-SY5Y cells with E2F8 knockdown or control were treated with DMSO or 10 μM SR9009 for 24 h and then infected with CVS-11 for 48 h. The protein levels of RABV-M, BMAL1, and GAPDH were analyzed by western blotting assay. **q** SH-SY5Y cells overexpressing EV, E2F8, E2F8-dD1, or E2F8-dD2 were infected with RABV for 48 h. The protein levels of p75NTR, RABV-M, FLAG-tagged proteins, and GAPDH were detected by western blotting assay. **r** ChIP-seq data from the GSM7503111 dataset were processed using the ChIPSeeker package in R (v4.4.2). Peaks associated with p75NTR were extracted and visualized utilizing the ggplot2 package. The stacked bar shows the percentage of total E2F8 binding sites in the defined distance bin (color-coded as indicated). The *x*-axis represents the binding site percentage (%) in the 5’ → 3’ direction, centered at the TSS. **s** E2F8-Flag CUT&Tag-qPCR results showing E2F8 enrichment in the promoter region of *p75NTR*. The data are presented as the mean ± SEM. Statistical significance was determined using independent-sample *t*-tests; *P < 0.05, **P < 0.01.

RABV relies predominantly on the nervous system for its transmission and replication, in contrast to HCMV, which is transmitted primarily via bodily fluids and disseminates to other organs following lytic infection in epithelial cells^61,62^. Furthermore, HCMV is human specific and does not naturally infect mice^62^, making RABV an ideal model for investigating the influence of the circadian clock on viral receptor expression and infection mechanisms both in vitro and in vivo. First, analysis of the GEO database revealed that p75NTR is the sole RABV receptor under circadian clock regulation (Fig. 3a; Supplementary Fig. S3a, b). Additionally, in REV-ERBα-KO SH-SY5Y cells, *p75NTR* mRNA levels were markedly reduced, accompanied by significant suppression of RABV entry (Fig. 3b, d); conversely, treatment with SR9009 or overexpression of REV-ERBα-FL significantly increased *p75NTR* mRNA levels and RABV entry, whereas the REV-ERBα-dDBD mutant failed to produce these effects (Fig. 3c, e; Supplementary Fig. S3c, d). The knockdown of p75NTR subsequently attenuated the enhancing effect of SR9009 on RABV infection and reduced the variability in cellular susceptibility to RABV across circadian cycles (Fig. 3f, g).

Next, we used a dual-luciferase reporter system to determine whether REV-ERBα influences the promoter activity of *p75NTR*. A significant increase in *p75NTR* promoter activity was observed with REV-ERBα-FL, whereas the REV-ERBα-dDBD mutant had no effect (Fig. 3h). Moreover, when both WT and REV-ERBα-KO HEK293 cells were transfected with the *p75NTR* promoter-luciferase reporter and then treated with DMSO or SR9009, the knockout of REV-ERBα abolished the potentiating effect of SR9009 on promoter activity (Fig. 3i). Collectively, these results indicate that REV-ERBα increases *p75NTR* promoter activity, thereby upregulating p75NTR expression and facilitating RABV infection.

Given that REV-ERBα is a transcriptional repressor that regulates gene expression by suppressing promoter activity^63^, we hypothesized that its effect on the *p75NTR* promoter was not direct but was mediated by another transcription factor. PROMO^64,65^ was used to predict transcription factors that bind to the *p75NTR* promoter. Given the nonrhythmic expression patterns of *GRM2* and *NCAM1* (Supplementary Fig. S3e), the predicted transcription factors linked to the promoters of *GRM2* and *NCAM1* were used as a filtering criterion. Ultimately, members of the E2F family, as well as AhR and SRF, were identified as potential regulators of p75NTR (Fig. 3j). The mRNA levels of these transcription factors were subsequently examined in SH-SY5Y cells treated with SR9009 or in cells with REV-ERBα KO. Notably, the expression of *E2F5*, *E2F7*, and *E2F8* was significantly downregulated following SR9009 treatment and correspondingly upregulated in REV-ERBα-KO SH-SY5Y cells (Fig. 3k). Furthermore, analysis of the GEO dataset GSE176393^54^ indicated that treatment of Calu-3 cells with SR9009 resulted in a pronounced decrease in *E2F8* mRNA levels (Supplementary Fig. S3f).

To elucidate the regulatory effect of E2F5, E2F7, and E2F8 on p75NTR, these transcription factors were overexpressed in SH-SY5Y cells, with only E2F8 overexpression leading to a significant decrease in the *p75NTR* mRNA level (Fig. 3l). Conversely, knockdown of E2F8 in SH-SY5Y cells resulted in a significant increase in *p75NTR* mRNA levels (Supplementary Fig. S3g). In addition, RNA-seq data from the GSE71574^66^ dataset revealed that E2F8 KO led to an increase in the mRNA level of *p75NTR* (Supplementary Fig. S3h). These findings support the hypothesis that E2F8 functions as a repressor of p75NTR transcription and that REV-ERBα increases *p75NTR* promoter activity by suppressing E2F8. To further validate this model, SH-SY5Y cell lines overexpressing E2F5, E2F7, and E2F8 were infected with CVS-11. Only E2F8 overexpression significantly inhibited RABV infection (Fig. 3m), whereas E2F8 knockdown increased RABV infection (Supplementary Fig. S3i). Additionally, when p75NTR expression was reduced in an SH-SY5Y cell line stably overexpressing E2F8, the inhibitory effect of E2F8 on RABV infection was abolished, indicating that E2F8 suppresses RABV infection primarily through modulation of p75NTR (Fig. 3n). Furthermore, HEK293 cells were cotransfected with a *p75NTR* promoter-luciferase reporter and REV-ERBα or E2F8. The results revealed that overexpression of E2F8 negated the REV-ERBα–mediated increase in promoter activity (Fig. 3o). In contrast, SR9009 treatment failed to promote RABV infection in an SH-SY5Y cell line in which E2F8 was knocked down (Fig. 3p).

To explore whether the DNA binding domains of E2F8 are essential for the regulation of p75NTR expression and RABV infection, we constructed E2F8 deletion mutants lacking DNA binding domain 1 (E2F8-D1) or DNA binding domain 2 (E2F8-D2). Our results demonstrated that both domains were critical for E2F8-mediated suppression of p75NTR expression and RABV infection (Fig. 3q), suggesting that E2F8 functions by directly binding to the *p75NTR* promoter. In support of this notion, analysis of ChIP-seq data from the GSE275631^67^ dataset in the GEO database revealed that in the *p75NTR* promoter (Fig. 3r), E2F8 peaks were located exclusively within 0–1 kb of the TSS (100%, purple). This complete proximal enrichment, with no peaks in distal regions, indicated that E2F8 bound directly to the core promoter elements of p75NTR, likely exerting strong and direct transcriptional control. Furthermore, CUT&Tag-qPCR experiments confirmed that E2F8 directly bound to the *p75NTR* promoter, thereby inhibiting its expression (Fig. 3s). These results demonstrate that E2F8 suppresses the promoter activity of *p75NTR* by binding to its promoter, whereas REV-ERBα increases the mRNA level of *p75NTR* by inhibiting E2F8 expression.

### E2F8 reciprocal feedback with REV-ERBα modulates host–virus interactions

**Fig. 4:**
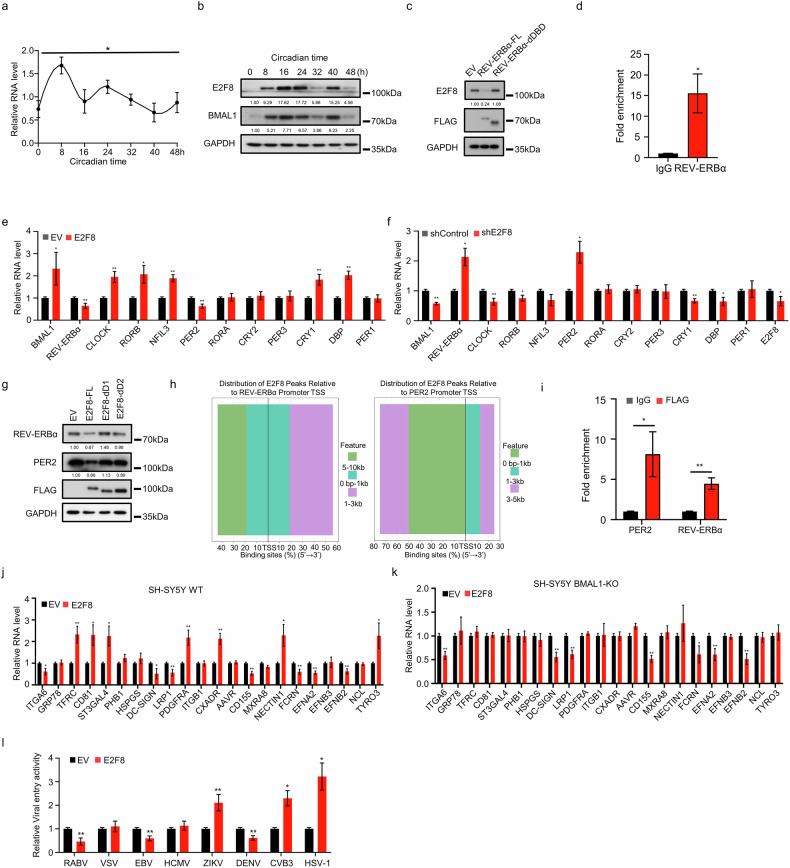
E2F8 reciprocal feedback with REV-ERBα modulates host–virus interactions. **a**, **b** Total RNA and protein lysates were extracted from SH-SY5Y cells synchronized with a 100 nM dexamethasone pulse. The mRNA levels of *E2F8* and *GAPDH* were measured by qRT-PCR (**a**), and the protein levels of E2F8, BMAL1, and GAPDH were assessed by western blotting assay (**b**). **c** Western blotting assay was used to measure the protein levels of E2F8, FLAG-tagged proteins, and GAPDH in SH-SY5Y cells overexpressing EV, REV-ERBα-FL, or REV-ERBα-dDBD. **d** REV-ERBα CUT&Tag-qPCR results showing enrichment of REV-ERBα in the promoter region of *E2f8*. **e**, **f** Total RNA was extracted from SH-SY5Y cells overexpressing EV or E2F8 (**e**) and from SH-SY5Y cells with E2F8 knockdown or negative controls (**f**). The mRNA levels of various clock components were measured by qRT-PCR. **g** SH-SY5Y cells overexpressing EV, E2F8, E2F8-dD1, or E2F8-dD2 were infected with RABV for 48 h. The protein levels of REV-ERBα, PER2, FLAG-tagged proteins, and GAPDH were analyzed by western blotting assay. **h**, **i** ChIP-seq data from the GSM7503111 dataset were processed using the ChIPSeeker package in R (v4.4.2). Peaks associated with REV-ERBα (left) and PER2 (right) were extracted and visualized utilizing the ggplot2 package. Stacked bars show the percentage of total E2F8 binding sites in defined distance bins (color-coded as indicated). The *x*-axis represents the binding site percentage (%) in the 5′ → 3′ direction, centered at the TSS (**h**). E2F8-Flag CUT&Tag-qPCR results showing E2F8 enrichment in the promoter region of REV-ERBα and PER2 (**i**). **j**, **k** WT (**j**) and BMAL1-KO (**k**) SH-SY5Y cells were infected with lentivirus carrying EV or E2F8 for 48 h. Total RNA was extracted, and the mRNA levels of neurotropic virus receptor genes were measured by qRT-PCR. **l** The effect of E2F8 overexpression on neurotropic virus entry into host cells was assessed in a manner similar to that described in Fig. 2a, b. All experiments were performed in triplicate. The data are presented as the mean ± SEM. Statistical significance was determined using independent-sample *t*-tests, whereas rhythmicity was evaluated by CFJHC. *P < 0.05, **P < 0.01.

The E2F8 protein functions as a negative regulator of the cell cycle, impeding G1 phase progression and stress responses through the modulation of gene transcription^68^. KEGG analysis of the peaks from the BMAL1 CUT&Tag-seq data revealed that the “cell cycle” pathway was enriched (Fig. 1d), prompting us to hypothesize that E2F8 is a clock-controlled gene (CCG). To investigate its relationship with the circadian clock, we analyzed an RNA-seq dataset from GSE234789, which revealed significant oscillations in *E2f8* mRNA levels across various Zeitgeber times in mouse submandibular gland tissues (Supplementary Fig. S4a). To confirm that E2F8 is a CCG, we evaluated its mRNA and protein levels via qRT‒PCR and western blotting assay at various circadian time points in synchronized SH-SY5Y cells or at different Zeitgeber times in brain tissues from mice entrained to a 12:12 light/dark cycle (Fig. 4a, b; Supplementary Fig. S4b, c). Ablation of BMAL1 or CRY1/2 results in arrhythmicity in cells or animals^60,69,70^. We examined the rhythmic pattern of E2F8 expression in BMAL1-KO SH-SY5Y cells and Cry1/2-DKO MEFs and found that when the cells were rendered arrhythmic, the oscillatory expression of *E2F8* was also abolished (Supplementary Fig. S4d, e). These findings demonstrated the dependency of E2F8 rhythmicity on the core circadian clock machinery. However, BMAL1 CUT&Tag-seq data revealed no binding peak in the *E2F8* promoter region (data not shown). Our previous experiments demonstrated that the clock factor REV-ERBα suppresses E2F8 expression, leading us to hypothesize that REV-ERBα drives its rhythmic expression. Indeed, the reduction in E2F8 protein levels induced by REV-ERBα depended on its DNA-binding domain (Fig. 4c). To determine whether this regulation is direct, we analyzed the public ChIP-seq dataset GSM8123058 and performed CUT&Tag-qPCR experiments, which confirmed that REV-ERBα bound to the promoter region of *E2F8* (Fig. 4d; Supplementary Fig. S4f).

Given that the cell cycle is in a state of dynamic equilibrium, we hypothesize the presence of a negative feedback regulatory mechanism between E2F8 and REV-ERBα, which may indirectly influence the circadian clock system. Surprisingly, SH-SY5Y cells with overexpressed or knockeddown E2F8 showed significant alterations in the mRNA levels of several circadian clock control factors, including *BMAL1, REV-ERBα, CLOCK, RORB, PER2, CRY1* and *DBP* (Fig. 4e, f). Notably, only the expression of *REV-ERBα* and *PER2* was downregulated following E2F8 overexpression. To elucidate this mechanism, we overexpressed E2F8-FL and mutants lacking DNA-binding domains in SH-SY5Y cells and found that the downregulation of REV-ERBα and PER2 expression required intact E2F8 DNA-binding domains, suggesting direct transcriptional repression (Fig. 4g). To explore whether E2F8 directly binds to the promoter regions of *REV-ERBα* and *PER2*, we mined publicly available ChIP-seq datasets for E2F8 (GSE275631) and analyzed the genomic distribution of E2F8 peaks relative to the TSS of the *REV-ERBα* and *PER2* promoters using ChIPseeker and custom R scripts. We found that the *REV-ERBα* promoter had a broader distribution: the largest proportion (~55%, green) resided 5–10 kb from the TSS (distal, likely enhancer regions), ~30% (cyan) was within 0–1 kb (proximal promoter), and ~15% (purple) was within 1–3 kb. In contrast, the *PER2* promoter was more proximally biased: ~45% (purple) was within 3–5 kb, ~30% (cyan) was within 1–3 kb, and ~15% (green) was within 0–1 kb, with minimal peaks farther away (Fig. 4h). Collectively, these data demonstrated the direct occupancy of E2F8 at the promoter regions of REV-ERBα and PER2, with a proximal predominance for PER2 and the inclusion of distal enhancers for REV-ERBα. Finally, using CUT&Tag-qPCR, we demonstrated that E2F8 bound directly to the promoter regions of *REV-ERBα* and *PER2* (Fig. 4i). These results suggest that E2F8 is a potential regulator of the circadian clock.

We further hypothesized that E2F8 influences the host circadian clock, thereby affecting the expression of neurotropic virus receptors. RNA-seq analysis of the GEO dataset GSE52157^71^ revealed that E2F8 deficiency in the mouse spleen significantly altered the expression of receptors for various neurotropic viruses (Supplementary Fig. S4g). The increase in these receptors due to E2F8 overexpression was linked to its effect on the circadian clock, as this increase was abolished following BMAL1 knockout (Fig. 4j, k). Conversely, the decrease in the level of receptor genes due to E2F8 overexpression resulted from its direct binding to their promoters, which persisted despite BMAL1 knockout (Fig. 4j, k). Finally, we demonstrated that E2F8-mediated regulation of receptor expression functionally affected neurotropic virus entry (Fig. 4l). In conclusion, our findings reveal an unconventional role for the cell cycle regulator E2F8 as a key component in a reciprocal feedback loop with the core circadian clock, thereby influencing host susceptibility to neurotropic viruses through both direct and indirect transcriptional mechanisms.

### Neurotropic RABV induces BMAL1 protein loss to compromise circadian clock stability

**Fig. 5:**
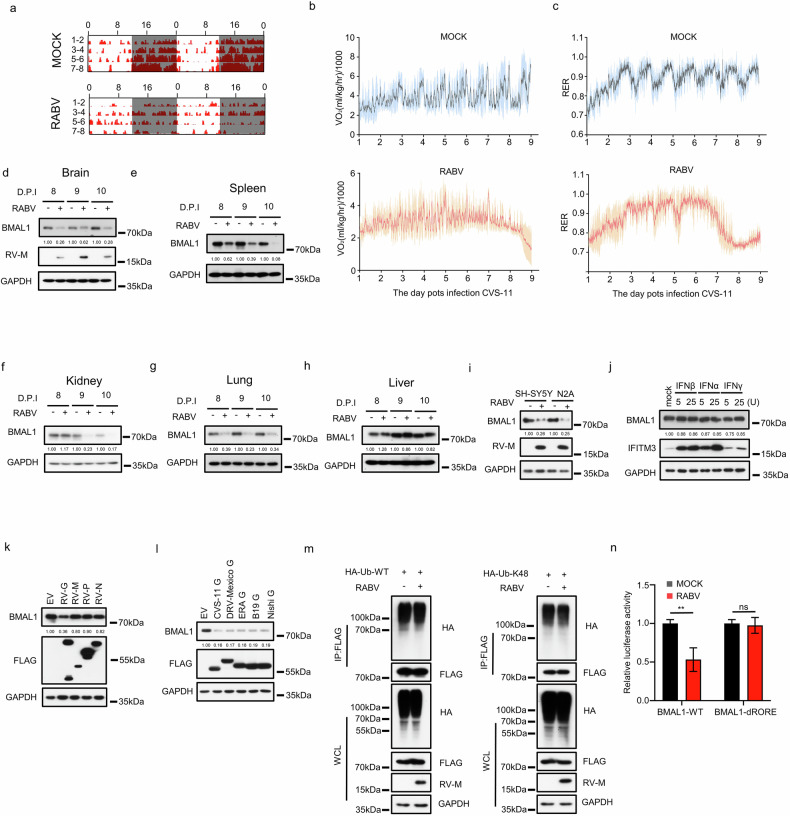
RABV disrupts the host circadian clock by downregulating expression of the BMAL1 protein. **a–c** Male mice (12 weeks old) were divided into RABV-infected (CVS-11 strain) and mock-infected (PBS) groups. Running wheel activity (**a**), oxygen consumption (**b**), and the respiratory exchange ratio (RER) (**c**) were monitored post-infection using the Lab Animal Monitoring System. **d–h** On Days 8, 9, and 10 post-RABV infection, mice from both the experimental and mock groups were euthanized, and tissues from the brain (**d**), spleen (**e**), kidney (**f**), lung (**g**), and liver (**h**) were collected. The protein levels of Bmal1, RABV-M, and GAPDH in these tissues were analyzed by western blotting assay. **i** Western blot analysis was performed on total protein extracts from SH-SY5Y and N2A cells infected with CVS-11 or mock-infected, and the expression of BMAL1, RABV-M, and GAPDH was assessed. **j** SH-SY5Y cells were treated with IFNβ, IFNα, or IFNγ (5 U or 25 U) for 16 h. Western blotting assay was used to detect BMAL1, IFITM3, and GAPDH in total protein extracts. **k**, **l** Western blot analysis of total protein from SH-SY5Y cells stably expressing FLAG-tagged CVS-11 proteins (G, M, P, N) (**k**) or various RABV G proteins (**l**) to assess the levels of BMAL1, FLAG-tagged proteins, and GAPDH. **m** HEK293 cells were co-transfected with Flag-tagged BMAL1 and either HA-tagged WT-Ub (left) or HA-tagged K48-only Ub (right). At 24 h post-transfection, cells were infected with CVS-11 for 48 h. Cell lysates were immunoprecipitated with anti-Flag antibody and immunoblotted with the indicated antibodies. **n** HEK293 cells were transfected with *BMAL1* promoter (FL/dRORE) luciferase reporters and infected with CVS-11. Luciferase activity was quantified at 48 h post-infection and normalized to that of the controls. All experiments were performed in triplicate. The data are presented as the mean ± SEM. Statistical significance was determined using independent-sample *t*-tests; **P < 0.01, ns not significant.

The mutual interaction between viruses and hosts is a dynamic process^72^. Previous research has shown that HSV-1 infection modulates *BMAL1* promoter activity^46^, suggesting that viral infections may disrupt host circadian rhythms. However, behavioral evidence from animal models and the underlying molecular mechanism remain scarce. To investigate in detail the impact of neurotropic virus infection on the host circadian clock, we chose RABV as our model because its infection can affect the host’s nervous system^73^. We examined the rhythmic patterns of integrated behavioral and metabolic functions in mock- and RABV-infected mice. Our analysis revealed that food intake did not differ significantly between RABV-infected and mock-infected mice prior to the onset of encephalitis and paralysis in the RABV group (Supplementary Fig. S5). However, the RABV-infected mice displayed arrhythmic patterns in wheel running activity (Fig. 5a), oxygen consumption (Fig. 5b), and the respiratory exchange ratio (Fig. 5c), indicating that RABV disrupts the host’s circadian clock.

Previous research has shown that BMAL1 is the only clock gene whose disruption of homeostasis results in circadian clock dysfunction^74^. To elucidate the molecular mechanisms underlying RABV-induced circadian rhythm disruption, we assessed changes in BMAL1 protein levels across multiple organs — the brain, spleen, kidney, lung, and liver — at various stages of viral infection. Our findings demonstrated that Bmal1 protein levels in the brain, spleen, and lung significantly decreased from the early to late stages of RABV infection. In contrast, BMAL1 levels in the kidney remained unchanged during the early stages, whereas those in the liver were unaffected throughout the infection (Fig. 5d–h). Notably, among these organs, RABV was detected exclusively in the brain, leading us to hypothesize that RABV disrupts the central clock, consequently impairing peripheral clocks.

Further investigation revealed a significant reduction in BMAL1 protein levels in SH-SY5Y and mouse neuroblastoma N2A cells cultured in vitro following RABV infection (Fig. 5i). Given that viral infections typically activate the host’s innate immune response, we explored whether this response could account for BMAL1 downregulation. Treatment of SH-SY5Y cells with varying concentrations of interferon-beta, interferon-alpha, and interferon-gamma did not alter BMAL1 levels (Fig. 5j), suggesting that viral components may directly modulate the host’s circadian clock.

To identify the specific viral factors involved, we overexpressed the G, M, P, and N proteins of RABV CVS-11 in SH-SY5Y cells. Owing to the large molecular weight of the L protein, we were unable to construct an overexpression plasmid. Our results indicated that only the RABV G protein significantly reduced BMAL1 protein expression (Fig. 5k). Moreover, G proteins from various RABV strains consistently decreased BMAL1 protein levels (Fig. 5l). The primary mechanisms leading to reduced BMAL1 protein levels were K48-linked polyubiquitination, which targets BMAL1 for proteasomal degradation^75,76^, and transcriptional repression of the *Bmal1* gene by REV-ERBα^34^. We found that RABV infection did not alter the levels of total ubiquitinated BMAL1 or K48-linked ubiquitinated BMAL1 (Fig. 5m). However, we discovered that RABV infection suppressed *BMAL1* promoter activity and that this repression was dependent on the presence of REV-ERBα binding sites. Specifically, a BMAL1 luciferase reporter mutant lacking these binding sites showed no response to RABV infection (Fig. 5n). These findings indicated that RABV infection disrupted the central circadian clock, leading to systemic perturbation of the host circadian system. The observed dysregulation of BMAL1 expression may be indirectly mediated by the effect of the viral G protein on REV-ERBα.

### RABV G protein targets HUWE1 to stabilize REV-ERBα via inhibition of K55/K59-specific K48 ubiquitination

**Fig. 6:**
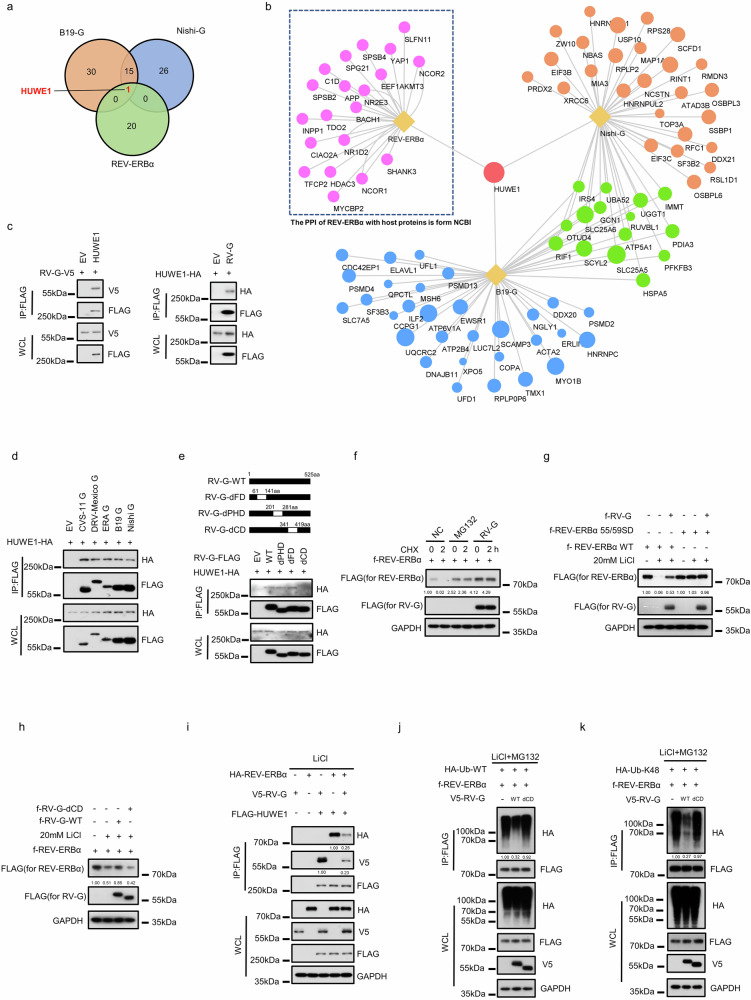
RABV G protein targets HUWE1 to stabilize REV-ERBα via inhibition of K55/K59-specific K48 ubiquitination. **a**, **b** A Venn diagram illustrates the overlap in protein‒protein interactions (PPIs) among Nishigahara G, B19 G, and REV-ERBα (**a**). A PPI network of the Nishigahara G, B19 G, and REV-ERBα proteins. Viral G proteins and REV-ERBα (baits) are shown in yellow. Node sizes correlate with the score from mass spectrometry analyses (**b**). **c** The binding of RABV G to HUWE1 was confirmed by reciprocal co-IP. **d** Interaction of G protein from various RABV strains fused with Flag to HUWE1-HA, as determined by Flag-IP and IB with anti-Flag and anti-HA antibodies. **e** Interaction of RABV G and mutants fused with Flag to HUWE1-HA, as determined by Flag-IP and IB with anti-Flag and anti-HA antibodies. **f** Immunoblot analysis of extracts from HEK293 cells transfected with REV-ERBα-FLAG and treated with MG132 (10 μM) or overexpressing CVS-11 G for 24 h, followed by treatment with cycloheximide (CHX; 25 μg/mL) for 0 h or 2 h. **g** Western blot analysis of WT-REV-ERBα-Flag or 55/59SD-REV-ERBα-Flag protein levels in HEK293 cells transfected with EV or CVS-11-G and then treated with 20 mM LiCl for 12 h. **h** Western blot analysis of WT-REV-ERBα-Flag protein levels in HEK293 cells transfected with EV/WT-CVS-11 G/dCD-CVS-11 G and then treated with 20 mM LiCl for 12 h. **i** V5-tagged CVS-11 G and HA-tagged REV-ERBα were cotransfected with FLAG-tagged HUWE1 into HEK293 cells, after which the cells were lysed and immunoprecipitated with anti-FLAG antibody and blotted with the indicated antibodies. **j**, **k** HA-tagged WT-Ub (**j**) or K48-specific-Ub (**k**) and Flag-tagged REV-ERBα were cotransfected with V5-tagged WT-CVS-11 G or dCD-CVS-11 G and then treated with 20 mM LiCl and 10 μM MG132 for 12 h. The cells were then lysed and immunoprecipitated with anti-FLAG antibody and blotted with the indicated antibodies.

To investigate the relationship between RV-G and REV-ERBα, we utilized the protein interaction network provided in the REV-ERBα record from the NCBI Gene database, which catalogs known interaction partners on the basis of curated experimental data. Previously, we mapped the protein‒protein interaction (PPI) profiles of G proteins from the SAD B19 and Nishigahara strains with host proteins in SH-SY5Y cells using affinity tag purification-mass spectrometry (AP-MS)^77^. However, no overlap was observed between these G protein PPI profiles and those of REV-ERBα (data not shown). After analyzing the proteins that interacted with REV-ERBα, we speculated that the low basal expression levels of certain proteins in neuronal cells might hinder the capture of the existing interactions. Given this, we conducted AP-MS analysis on HEK293 cell lysates following co-immunoprecipitation (co-IP) with 3× Flag-tagged G proteins from the RABV strains Nishigahara and SAD B19. These analyses revealed 42 high-confidence interactions for Nishigahara G and 46 for SAD B19 G. By integrating these data with the 21 known REV-ERBα-interacting host proteins from the NCBI database, we constructed an interactome map (Fig. 6b; Supplementary Tables S2–S4). Notably, the E3 ubiquitin ligase HUWE1 emerged as the sole protein common to all three interactomes (Fig. 6a, b). Subsequent reciprocal assays confirmed the interaction between RV-G and HUWE1 (Fig. 6c). This interaction was conserved across G proteins from different RABV strains (Fig. 6d), suggesting its consistency across viral variants. The RV-G protein comprises three functional domains: the fusion domain (FD), central domain (CD), and pleckstrin homology domain (PHD)^78^. To determine which domain mediates the interaction with HUWE1, we generated RV-G mutants lacking each domain individually. Deletion of the CD domain abolished the RV-G–HUWE1 interaction (Fig. 6e).

REV-ERBα is known to undergo lithium-stimulated degradation, a process mediated by the HUWE1/PAM complex through K48 ubiquitination at serines 55 and 59^79^. Consistent with prior reports, we demonstrated that the proteasome inhibitor MG132 extended the half-life of REV-ERBα. Similarly, overexpression of RV-G prolonged the half-life of REV-ERBα (Fig. 6f). To further explore this mechanism, we engineered a REV-ERBα mutant mimicking phosphorylation at serines 55 and 59 (REV-ERBα-55/59 SD). Treatment with 20 mM LiCl reduced the protein levels of WT REV-ERBα but not those of the REV-ERBα-55/59 SD mutant. Moreover, RV-G inhibited the lithium-induced degradation of REV-ERBα (Fig. 6g). However, RV-G mutants incapable of interacting with HUWE1 failed to prevent LiCl-induced REV-ERBα degradation (Fig. 6h).

On the basis of these findings, we hypothesized that RV-G competes with REV-ERBα for binding to HUWE1. Co-IP experiments revealed that LiCl treatment increased the interaction between REV-ERBα and HUWE1, an effect that was suppressed by RV-G (Fig. 6i). Furthermore, WT RV-G inhibited both total ubiquitination and K48-specific ubiquitination of REV-ERBα, an effect contingent upon its ability to bind HUWE1 (Fig. 6j, k). Finally, we sought to determine whether the mechanism by which RABV disrupts the circadian clock is universal. We found that the G proteins of VSV and EBOV did not significantly affect the half-life of REV-ERBα or its Li⁺-induced degradation (Supplementary Fig. S6a, b), indicating that virus–host interactions with the circadian clock are complex and diverse. Collectively, our results demonstrate that RV-G competes with REV-ERBα for binding to HUWE1, thereby attenuating K48 ubiquitination at serines 55 and 59 of REV-ERBα. This competition stabilizes REV-ERBα, leading to its accumulation, which in turn suppresses BMAL1 expression and disrupts the host’s circadian rhythm.

### Effects of time of day, chronic jet lag, and REV-ERBα knockout on the susceptibility of the host to the neurotropic virus RABV

**Fig. 7:**
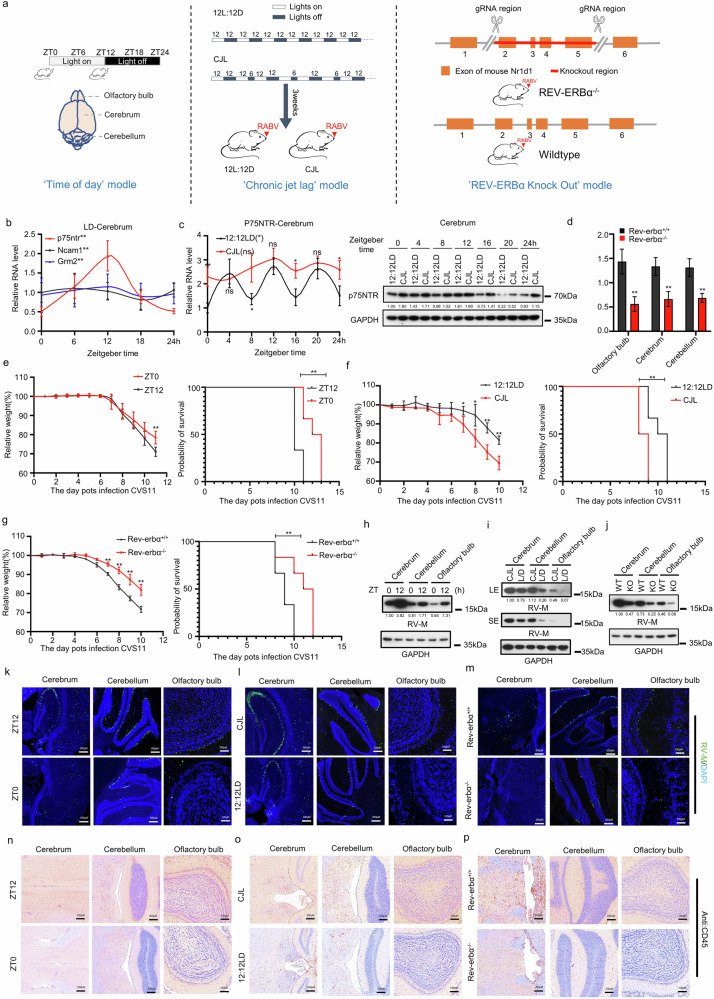
Effects of time of day, chronic jet lag, and REV-ERBα knockout on the susceptibility of the host to the neurotropic virus RABV. **a** Schematic diagram illustrating the strategy for constructing three animal models: “Time of Day”, “Chronic Jet Lag (CJL)”, and “Rev-ERBα Knockout”. **b** qRT-PCR was used to measure the mRNA levels of *p75NTR* in the brains of the “Time of Day” model at different Zeitgeber times. Data are presented as the mean ± SEM (*n* = 5). **c** Samples were collected at multiple Zeitgeber times from CJL mice or 12:12 L/D mice. The mRNA and protein levels of p75NTR were measured by qRT-PCR and western blotting assay, respectively. In the mRNA curve, relative expression was normalized to the mean *p75NTR* mRNA level at ZT0 in 12:12 L/D mice. The data are presented as the mean ± SEM (*n* = 5). **d** qRT-PCR was used to measure the mRNA levels of *p75NTR* in the brains of WT/Rev-ERBα-KO mice at different Zeitgeber times, and the data are presented as the mean ± SEM (*n* = 5). **e**–**g** Body weight changes (left) and Kaplan‒Meier survival curves (right) of C57BL/6 J mice following intranasal infection with CVS-11. **e** Time-of-day experiment: Mice were infected either at ZT0 (lights-on) or ZT12 (lights-off) under standard 12:12 light/dark conditions. **f** CJL mice/12:12 L/D mice infected with CVS-11 and subsequently maintained under either control 12:12 L/D conditions or chronic jet lag conditions. **g** WT and Rev-ERBα knockout mice infected with CVS-11 under standard 12:12 L/D conditions (body weight *n* = 5; survival *n* = 12). **h**, **k**, **n** Time of Day group. On Day 10 after RABV infection, mice from the “Time of Day” group (ZT0 vs ZT12) were analyzed using three complementary approaches. **h** Brain regions, including the olfactory bulb, cerebellum, and cerebrum, were dissected and homogenized for protein extraction; western blotting assay was performed to measure the level of the RABV M protein, with GAPDH used as the loading control. **k** Parallel cohorts were perfused with PBS and fixed with formaldehyde; cryosections were subjected to immunofluorescence staining to visualize the distribution of the RABV M protein. **n** Additional paraffin-embedded sections were processed for immunohistochemical staining to quantify CD45^+^ immune cell infiltration in the same brain regions. **i**, **l**, **o** CJL group. On Day 10 after RABV infection, the mice in the “CJL” group (CJL vs 12:12 L/D) were subjected to western blotting assay, immunofluorescence staining, and immunohistochemical staining. **i** Protein extracts from the olfactory bulb, cerebellum, and cerebrum were analyzed for RABV M protein expression, with GAPDH used as a control. **l** PBS-perfused and formaldehyde-fixed cryosections were subjected to immunofluorescence staining to detect RABV M protein localization. **o** Paraffin-embedded sections were subjected to immunohistochemical staining to assess CD45^+^ cell infiltration in the indicated brain regions. **j**, **m**, **p** Rev-ERBα-KO. On Day 10 after RABV infection, mice from the “Rev-ERBα knockout” group (WT vs Rev-ERBα-KO) were evaluated by western blot assay, immunofluorescence staining, and immunohistochemical staining. **j** Protein extracts from the olfactory bulb, cerebellum, and cerebrum were subjected to western blotting assay to detect the RABV M protein, with GAPDH used as a reference. **m** Cryosections prepared after PBS perfusion and formaldehyde fixation were subjected to immunofluorescence staining to visualize the RABV M protein. **p** Paraffin-embedded sections were processed for immunohistochemical staining to quantify CD45^+^ immune cells in the same brain regions. The data are from three independent experiments. Log-rank test (Kaplan‒Meier survival curves), ARS (rhythmicity) and independent-samples *t*-test, *P < 0.05, **P < 0.01, ns not significant.

Despite sharing the same fundamental molecular clockwork, the circadian system in monolayer-cultured cells differs significantly from that in living animals^80,81^. To examine the influence of the circadian clock on neurotropic virus infections in vivo, we selected RABV as a representative neurotropic virus and utilized C57BL/6 mice as the experimental model. A standard 12 h light and 12 h dark cycle (12:12 L/D) was used to establish a “time of day” research model. Additionally, 6-h light-phase advances were performed every 2 days^82^ to induce chronic circadian disruption, whereas CRISPR-Cas9 technology was used to generate a “Rev-erbα knockout” model (Fig. 7a). Mice were infected with the CVS-11 strain of RABV via intranasal administration, and their brains were subsequently dissected into the olfactory bulb, cerebrum, and cerebellum to assess viral infection. Daily fluctuations in RABV receptor and clock factor expression were quantified in brain tissue harvested every 6 h from mice entrained to a 12:12 L/D cycle. The rhythmic expression of the clock genes *Bmal1* and *Rev-erbα* in the olfactory bulb, cerebrum, and cerebellum confirmed the efficacy of our “Time of Day” model (Supplementary Fig. S7a–c). Consistent with our cellular-level findings, only the mRNA expression of *p75NTR* exhibited a daily rhythm, peaking at ZT12 (Fig. 7b; Supplementary Fig. S7d, e). A defining feature of circadian rhythms is their persistence in the absence of environmental cues. The sustained rhythmic expression of *Bmal1* and *Rev-erbα* mRNA in the three brain regions under constant darkness (CD) confirmed this characteristic (Supplementary Fig. S7f–h), with the *p75NTR* rhythm similarly persisting across these regions (Supplementary Fig. S7i–k).

In the chronic jet lag (CJL) model, mice maintained under a 12:12 L/D cycle served as controls. By monitoring rhythmic behavior and metabolic activity, we confirmed that the “repeated 6 h light phase advances every two days to induce chronic circadian disruption” regimen effectively disrupted the circadian clock in CJL mice (Supplementary Fig. S8a, b). Moreover, the mRNA levels of the clock genes *Bmal1* and *Rev-erbα* lost their rhythmicity in the three brain regions of CJL mice (Supplementary Fig. S8c–e). We subsequently confirmed that the rhythmicity of p75NTR expression was also abolished and that the mRNA and protein levels of p75NTR in the olfactory bulb, cerebellum, and cerebrum of CJL and 12:12 L/D mice displayed dynamic changes across the Zeitgeber time axis, with significant differences observed at most Zeitgeber time points (Fig. 7c; Supplementary Fig. S8f, g). In Rev-erbα-KO mice, despite the critical role of REV-ERBα in circadian rhythms, no substantial changes were observed in the period or amplitude of rhythmic behavior following Rev-erbα knockout (Supplementary Fig. S9), which aligns with the findings of previous studies^83^. Consistent with our cellular-level results, the mRNA levels of the RABV receptor p75NTR were significantly lower in Rev-erbα-KO mice than in WT mice (Fig. 7d).

We first conducted infection experiments using the “Time of Day” model. Compared with those infected at ZT0 (the onset of the inactive/light phase), mice infected at ZT12 (the onset of the active/dark phase) exhibited more rapid weight loss (Fig. 7e) and reached the disease endpoint (score of 5) earlier (Supplementary Fig. S10a). Upon reaching the clinical endpoint, the mice were euthanized (Fig. 7e). Subsequent analysis of RABV replication kinetics in the mouse brain revealed that the virus initially replicated in the cerebrum approximately 4–6 days post-infection (d.p.i.) before it spread to the olfactory bulb and cerebellum at later stages (8–10 d.p.i.). Notably, at all the measured time points and across all the brain regions, the viral load and replication rate were significantly greater in mice infected at ZT12 compared with those infected at ZT0 (Supplementary Fig. S10b). This finding suggests that viral amplification is more pronounced at ZT12. As anticipated, by the 10th d.p.i., a greater abundance of RABV M protein was detected at ZT12 (Fig. 7h), accompanied by an increased number of viral particles in the brain, compared with those detected at ZT0 (Fig. 7k). Following infection with the rabies challenge strain, the mice developed pathogenic encephalitis. To evaluate the extent of encephalitis induced by RABV infection, CD45-positive cells were identified via immunohistochemical staining, and the levels of interferon β and γ, proinflammatory cytokines, inflammasome activation, and chemokines were quantified by qPCR. CD45-positive cells were more abundant in the brains of mice infected at ZT12 than in those of mice infected at ZT0 (Fig. 7n). Additionally, the mRNA levels of all the immune-related genes assessed were elevated in ZT12 mice compared with ZT0 mice (Supplementary Fig. S10c), indicating that the inflammatory immune response was more robust in the brains of ZT12 mice. Given that circadian rhythms modulate host immune responses, we examined whether antiviral immunity in the mouse brain differs between ZT0 and ZT12 under basal conditions, potentially explaining the greater susceptibility to RABV at ZT12. Analysis of IFNβ secretion and interferon-stimulated gene (ISG) expression revealed no significant differences between ZT0 and ZT12 in the resting state (Supplementary Fig. S10d, e). Collectively, these results suggest that RABV infection is modulated by the time of day, which correlates with the regulation of the RABV receptor p75NTR by the biological clock.

Three weeks after initiating the CJL light-dark regimen, both 12:12 L/D and CJL mice were inoculated with RABV. Compared with their 12:12 L/D counterparts, CJL mice exhibited weight loss and initial clinical symptoms earlier, with accelerated weight loss, faster disease progression, and higher mortality rates (Fig. 7f; Supplementary Fig. S11a). Consistent with the observed disease progression, CJL mice displayed elevated viral loads in the cerebrum, cerebellum, and olfactory bulb (Fig. 7i, l), along with more intense inflammation and increased mRNA levels of immune-related factors (Fig. 7o; Supplementary Fig. S11b–d). Research has indicated that CJL can impair immune function and exacerbate vulnerability to pathogens^84–86^; for instance, CJL alters the mouse lung transcriptome, downregulating the expression of immune regulatory genes while upregulating the expression of transcripts that promote viral replication and immune evasion^87^. Nonetheless, reports elucidating the effect of CJL on antiviral immune responses within the host central nervous system are lacking. To investigate whether CJL affects antiviral immune responses in the mouse brain, we measured IFN-β secretion and ISGs expression at different Zeitgeber times (ZT0, ZT4, ZT8, ZT12, ZT16, ZT20, and ZT24) in CJL and 12:12 L/D mice. We observed that, under basal conditions, compared with those in 12:12 L/D mice, IFN-β secretion and expression of ISGs in the brains of CJL mice were persistently modestly reduced (Supplementary Fig. S11e, f). These data suggest that the increased susceptibility of CJL mice to RABV results from a combined effect of impaired antiviral immune responses in the brain and attenuated oscillatory expression of the RABV receptor p75NTR, which remains elevated at most Zeitgeber times relative to mice under regular light-dark cycles. Given that IFN-β secretion and expression of ISGs in CJL mice were only modestly lower than those in 12:12 L/D mice, we propose that the differences in p75NTR rhythmicity are likely the dominant contributing factor.

Finally, in RABV challenge experiments involving WT and Rev-erbα-KO mice, we found that the suppression of *p75NTR* expression in Rev-erbα-KO mice impeded RABV infection, prolonged the incubation period, and slowed disease progression (Fig. 7g; Supplementary Fig. S12a). Rev-erbα-KO mice exhibited reduced viral loads in the cerebrum, cerebellum, and olfactory bulb (Fig. 7j, m), less severe inflammation and lower mRNA levels of immune-related factors (Fig. 7p; Supplementary Fig. S12b–d). In summary, our findings demonstrate that the timing of RABV infection critically influences its outcome. Disruption of the circadian clock exacerbates disease progression following RABV infection, whereas loss of the clock factor REV-ERBα results in partial resistance to RABV, thereby extending host survival (Supplementary Fig. S13).

## Discussion

The circadian clock is a fundamental regulator of physiological processes, exerting profound effects on immunity, metabolism, and hormone regulation across both in vitro and in vivo systems^88,89^. The interaction between the circadian clock and pathogens is intricate^90^, and previous studies have demonstrated time-of-day effects on viral infection and replication, primarily through the modulation of immune responses and other host factors^46,49^. This current study advances the field by extending the understanding of circadian control from immune modulation to include receptor-level rhythmicity and regulation of viral entry, specifically focusing on its role in modulating receptors that are essential for viral entry of viruses with neurotropic or neuroinvasive potential and establishing a correlation between their rhythmic expression and time-dependent host susceptibility to neurotropic viruses. CUT&Tag-seq analysis revealed prominent BMAL1 binding peaks in the promoter regions of most neurotropic viral receptors, indicating direct transcriptional regulation. Rhythmic receptor expression, driven by BMAL1 and REV-ERBα, was observed in SH-SY5Y cells, cerebral organoids, and mouse models. We found that host susceptibility to neurotropic viruses varied with time of day: susceptibility to RABV aligned with the rhythm pattern of REV-ERBα, whereas susceptibility to viruses such as HCMV, CVB3, and EBOV corresponded to the BMAL1 rhythm pattern. Subsequently, we demonstrated that mice infected with the RABV challenge strain CVS-11 at ZT12 exhibited significantly elevated encephalitis and viral load compared with those infected at ZT0, with these time-of-day effects suggesting that circadian gating of viral entry receptors (e.g., p75NTR) could inform chronotherapeutic strategies for rabies prophylaxis. Although previous studies have demonstrated that CJL leads to a defective immune response in mice^84,91^, the effect of CJL on cerebral immune function remains poorly characterized. Our data demonstrated that interferon-β secretion and proinflammatory cytokine levels in the brains of CJL mice were modestly reduced relative to those of mice maintained under a regular 12:12 light/dark cycle across most zeitgeber time points. More strikingly, we found that the rhythmic expression of the RABV receptor p75NTR was abolished in CJL mouse brains, resulting in elevated p75NTR mRNA and protein levels at most time points compared with those of the regularly entrained controls. The increased susceptibility of CJL mice to RABV therefore likely reflects the combined consequences of circadian disruption — namely, a mildly attenuated cerebral immune response and sustained upregulation of p75NTR — with the latter emerging as the primary driver. The heightened vulnerability in CJL models parallels human shift-work disorders, where chronic circadian disruption may exacerbate susceptibility to infections, while the protective phenotype in Rev-erbα-KO mice highlights REV-ERBα as a potential therapeutic target for modulating viral pathogenesis. On the basis of these results, it could be inferred that the timing of neurotropic virus infection in humans throughout the day may significantly influence disease severity or incubation period duration. This concept holds practical potential: vaccinating individuals at the circadian time of peak susceptibility to a specific virus may increase immune responses. Similarly, it is possible that synchronizing cell cultures for infection at peak susceptibility may increase viral titers, potentially reducing production costs.

The E2F family comprises a series of transcription factors that are primarily known for their role in regulating the cell cycle, with only E2F1 and E2F4 having been shown to participate in host–virus interactions^92,93^; however, the involvement of other family members in viral infections remains largely unexplored. This study revealed the first evidence that E2F8 modulates neurotropic virus receptors, either directly or indirectly, consequently impeding virus entry. Moreover, we elucidated a feedback loop between E2F8 and REV-ERBα and the transcriptional repression of PER2 by E2F8, which implies that E2F8 may serve as a putative regulator of circadian rhythms. Nevertheless, the partial functional redundancy of REV-ERBβ and PER1 may compensate for their loss, thereby maintaining the overall circadian rhythm^94,95^. Therefore, further research is needed to assess whether alterations in E2F8 affect the host circadian rhythm in vivo. Furthermore, given the complex network regulation of target genes by clock genes and E2F family members, additional studies should investigate whether other clock components influence E2F family members and the role of E2F family transcription factors in circadian rhythms.

Viral strategies to evade host immunity or accelerate replication are focused primarily on the suppression of immune functions^96^; however, disruption of the host circadian clock may also be a crucial mechanism enabling viruses to establish infection. We observed that RABV infection disrupted host circadian rhythms, altering BMAL1 homeostasis in the brain, spleen, kidney, and lung. Because RABV was detected only in the brain, this suggests that RABV disrupts the central clock, causing desynchronization with peripheral clocks in other organs. Previous studies have indicated that inflammatory mediators alter clock gene expression and SCN firing rhythms^97,98^. Given that attenuated RABV strains induce encephalitis^99^, brain inflammation likely drives this disruption. Curiously, despite the paralysis observed in the mice in the later stages of infection, liver Bmal1 homeostasis remained stable, indicating that there are other regulatory mechanisms in addition to metabolic entrainment that make liver metabolism more independent of the central clock than other organs are. More importantly, beyond inflammation-driven circadian disruption, we revealed that the RABV glycoprotein competitively binds to the E3 ubiquitin ligase HUWE1, inhibits REV-ERBα K55/K59-specific K48 ubiquitination, prevents REV-ERBα degradation via the ubiquitin‒proteasome pathway, and thereby destabilizes BMAL1 protein homeostasis. Finally, we speculate that although overexpression of the RABV G protein reduces BMAL1 levels in cells, the inherent complexity and resilience of the circadian system may buffer against rhythm disruption when RABV-G is expressed alone in mice. Instead, the circadian disturbances seen during RABV infection likely stem from the combined effect of virus-induced severe encephalitis and RABV-G-mediated perturbation of clock gene protein homeostasis.

Overall, our work establishes the circadian clock as a central regulator of neurotropic virus–host interactions and provides new insights into how pathogens disrupt the host’s circadian rhythm and the potential role of the E2F family in circadian clock regulation. By mapping precise clock–virus interfaces, we highlight the opportunities for chrono-pharmacological intervention and the importance of maintaining a regular lifestyle.

## Materials and methods

### Mice

C57BL/6 N WT mice (8–12 weeks old, mixed sexes) were purchased from Wuhan Institute of Biological Products Co., Ltd. Rev-erbα heterozygous mice were procured from Cyagen Inc. (Suzhou, China) for breeding to generate Rev-erbα knockout mice and WT mice. All of the mice were fed rodent chow ad libitum. All the studies were approved by the Institutional Animal Care and Use Committee of Medical Research Institute, Wuhan University.

### Cerebral organoids

Induced pluripotent stem cells (iPSCs, purchased from SAIOS Biotech, Inc.) were cultured in a Matrigel-coated T25 flask with Essential 8 flex medium (Gibco, A2858501). On Day 0, the iPSCs were counted and seeded at 1 × 10^6^ cells/well in a six-well plate in Essential 8 Flex Medium supplemented with 10 μM ROCK inhibitor Y-27632 (MCE, HY-10071). On Day 1, embryonic bodies were formed, and the medium was replaced with E6 medium (50 mL of E8 basal + 0.1 μM LDN + 10 μM SB431542 + 2 μM XAV + 20 μg/mL insulin + 1% P/S). The medium was changed every two days until the iPSCs were induced into ectodermal cells (approximately 10 days), after which the medium was replaced with NI medium (50 mL of DMEM/F12 + 50 of neurobasal medium + 1% B-27 + 0.5% N-2 + 1× GlutaMAX + 1% P/S), and the cells were cultured for 60 days. The entire culture process was carried out under gentle shaking on a shaker (80 rpm).

### Antibodies and reagents

Anti-PER2 (ABclonal, A5107), anti-p75NTR (ABclonal, A19088), anti-E2F8 (ABclonal, A1135), anti-BMAL1 (Cell Signaling Technology, D2L7G), anti-REV-ERBα (Cell Signaling Technology, E1Y6D), anti-HCMV-PP65 (gift from Dr. Minhua Luo), anti-RABV-M (gift from Dr. LingZhao), anti-Flag (Sigma‒Aldrich, F3165), anti-HA (Abcam, ab9110), anti-V5 (Proteintech, 14,440–1-AP), and anti-GAPDH (ABclonal, AC033) antibodies were used. SR9009 (MedChemExpress, HY-16989), Lipofectamine™ 2000 Transfection Reagent (Invitrogen 1,679,991), MG132 (Sigma‒Aldrich, C2211), CHX (MedChemExpress, HY-12320), and LiCl (MedChemExpress, HY-Y0649) were obtained from the indicated suppliers.

### Plasmids

*REV-ERBα*-Flag (gene ID: 9572), E2F8-Flag (ge ne ID: 79733), E2F7*-*Flag (gene ID: 36270), E2F5-Flag (gene ID: 1875), and related mutants were inserted into the pQCXIP retroviral vector (deposited by Martin Dorf). p75NTR-luc, BMAL1-luc, and related mutants were inserted into the PGL4 vector (deposited by Martin Dorf). The plasmids described above were constructed for this study. HUWE1-Flag, HUWE1-HA, VSV-G-Flag, Ebola virus-G, CVS 11-P-Flag, CVS 11-M-Flag, CVS 11-N-Flag, CVS 11-G-Flag, ERA-G-Flag, B19-G-Flag, Nishigahara-G-Flag, DRV-Mexico-G-Flag, Ub-WT-HA, and Ub-K48-HA plasmids were preserved in the laboratory.

### Viruses and infection experiments

HCMV was a gift from Dr. Minhua Luo. Coxsackievirus B3 was a gift from Dr. Yin Wu. HSV-1 was a gift from Dr. Junjie Zhang. RABV, Zika virus, and DENV were preserved in the laboratory. The pseudoviruses were constructed through a lentiviral packaging system, as previously described^77^. Cells for various infection assays were seeded in 24-well plates. When the cell density reached 90% in both the experimental and control groups, RABV or HCMV was added at a multiplicity of infection (MOI) of 0.1 or 5, respectively. Following a 48 h incubation period post-infection, the cells were harvested to assess the viral load via western blot analysis. In the viral entry assay, cells were cultivated in 24-well plates to 90% confluency. Viruses were added at an MOI of 10 and incubated at 4 °C for 1 h to allow sufficient attachment to the cell surface. Nonadherent viruses were removed by washing with PBS, followed by a 1 h incubation at 37 °C. Cells were then collected, and viral genome abundance was detected by qPCR. For all the RABV infection experiments involving animals in this study, the animals were anesthetized with isoflurane. The RABV strain CVS-11, with a titer of 10^3^ TCID_50_ in 25 μL, was administered intranasally to mice as described by Rosseels et al.^100^. At the required time points after viral infection, the mice were euthanized, and mouse brain tissue was collected for qPCR, western blotting assay, immunofluorescence staining, and immunohistochemistry analysis of virus abundance and brain tissue inflammation.

### Regular light-dark cycles

For daily cycle experiments, WT mice were maintained under constant environmental conditions and entrained to a 12 h light/12 h dark cycle (light period from 8:00 a.m. to 8:00 p.m.) for at least 2 weeks before the experiments. The animals were euthanized at 8:00, 14:00, 20:00, and 2:00 the next day and at 24:00, corresponding to ZT0, ZT6, ZT12, ZT18, and ZT24, respectively. For experiments conducted under constant darkness, the animals were kept in constant darkness for 3 days prior to sampling at CT0, CT6, CT12, CT18, and CT24. After euthanasia, the brain tissues were collected for further analysis of the rhythmic changes in circadian clock factors and viral receptors. REV-ERBα-KO mice, as well as their WT littermates, were subjected to a regular light-dark cycle (12 h light:12 h dark) for more than two weeks. At ZT12 (20:00), which has been reported as the time point of maximal REV-ERBα activity at promoter sites^101^, intranasal viral infection was initiated.

### CJL conditions

To establish a chronic jet lag model in mice, we progressively advanced the light-dark cycle of the mouse housing environment by 6 h every 2 days, while the control group of mice remained under a regular light-dark cycle of 12 h each. This altered lighting schedule was maintained for a minimum of three weeks. Concurrent with the final light-phase advancement, both the CJL model and control groups of mice were infected with CVS-11.

### Monitoring of mouse circadian behavior

A Comprehensive Lab Animal Monitoring System (CLAMS; Columbus Instruments) was utilized to assess the wheel-running activity, metabolism, and feeding behavior of the mice. All monitored mice were individually housed in cages with ad libitum access to water and food. For experiments involving the effect of virus on mouse circadian rhythms, mice were maintained under a regular 12 h light and 12 h dark cycle, and the experimental endpoint was determined by the loss of locomotor activity following virus-induced encephalitis. In the behavioral monitoring experiments of the CJL model mice, the environmental lighting conditions were consistent with those mentioned above, whereas the WT mice were housed under standard regular lighting conditions.

### CUT&Tag-seq

The experiments were conducted according to the instructions provided by Vazyme (TD904). In brief, cerebral organoids were digested into single cells using Cell Recovery Solution for Organoids (Yeasen, 41421ES60), and approximately 2.5 × 10^5^ cells were collected. The dissociated cells were cross-linked with Enhancer buffer, combined with activated ConA beads and incubated overnight with anti-BMAL1 antibodies. Following incubation, the supernatant was removed, and the samples were incubated with a secondary antibody (Vazyme, Ab207) for 1 h at room temperature with rotation. The supernatant was subsequently discarded, and the ConA beads were incubated with pA/G-Tnp Pro and TTBL, each for 1 h at both room temperature and 37 °C. After TTBL incubation, 2 mL of 10% SDS was added to each sample and incubated at 55 °C for 10 min. The fragmented DNA in the supernatant was enriched using activated DNA Extract Beads Pro, and a next-generation sequencing library was constructed according to the manufacturer’s protocol (Vazyme, TD202). Sequencing was performed on the Illumina NovaSeq platform by Frasergen Bioinformatics Co., Ltd.

### CUT&Tag-qPCR

The precipitated chromatin DNA samples were obtained with a Hyperactive Universal CUT&Tag Assay Kit (TD904, Vazyme), in which the fragmented DNA was released from the DNA extract beads using CUT&Tag stop buffer (TD904-C1), followed by qPCR to quantify the abundance of the target gene. The samples were analyzed on a Bio-Rad CFX96 real-time PCR system using Taq Pro Universal SYBR qPCR Master Mix (Vazyme). The enrichment fold change of the REV-ERBα and E2F8 binding sites relative to that of DNA spike-in was determined using threshold cycle values and the 2^−ΔΔCt^ method.

### Data mining from the GEO database

We acquired the GSE176393, GSE248721, GSE222744, GSE71574, GSE52157, GSE234789, GSM7503111 and GSM8123058 datasets, which contain RNA-seq data or ChIP-seq data (GSM7503111 and GSM8123058) from diverse cell types and murine tissues that are pertinent from the public repository of the GEO website. The narrowPeak format files from the GSM7503111 dataset were processed using the ChIPSeeker package, and the bigWig format file of the GSM8123058 dataset was processed using the rtracklayer and GenomicRanges packages; the results were visualized with the ggplot2 package. A gene was defined as a differentially expressed gene (DEG) when there was a statistically significant difference in the transcription level of a gene in different test environments. The DEGs were identified from the long-expression values using the LIMMA package with Benjamini‒Hochberg correction to control the rate of false discovery and DESEq2 in multiple testing options. Cutoff criteria (*P* value < 0.05 and |logFC| ≥ 1.0) were applied to detect significant DEGs from all the datasets. All big data analyses were conducted using the R programming language (v4.4.2).

### Statistical analyses for rhythmicity assessment

All analyses were performed using R software (v4.5.2). For rhythmic gene fitting curves that exhibited approximate symmetry, autoregressive spectral estimation (ARS) was applied to assess rhythmic significance. Analyses were conducted in R using the MetaCycle package, which implements ARS alongside other rhythmicity detection algorithms. The ARS procedure was run via the meta2d() function, which specified the range of expected periods (minper and maxper) and a default period (ARSdefaultPer) within this range.

For rhythmic gene fitting curves that were asymmetric or based on sparse sampling points, we applied cosine fitting combined with joint significance testing of harmonic components (CFJHC) to evaluate rhythmicity. Specifically, we used the base R functions for linear modeling and the MASS package for multivariate normal sampling. The observed values were fitted against cosine and sine terms corresponding to the specified period (e.g., 24 h). The model provided estimates of amplitude (defined as the square root of the sum of squared cosine and sine coefficients), peak-to-trough difference (twice the amplitude), and phase (calculated as the arctangent of the ratio between sine and cosine coefficients). To account for uncertainty in amplitude, we generated repeated parameter samples from the joint distribution of the model coefficients using the mvrnorm() function in the MASS package and calculated the amplitude for each sample. To determine rhythmicity, amplitudes were compared with a predefined threshold derived from nonrhythmic genes. A one-sample *t*-test was performed using the base R’s t.test() function to evaluate whether the mean amplitude exceeded this threshold, and genes showing significant results were classified as rhythmic.

## Supplementary information

Supplementary Information

Supplementary Table S1

Supplementary Table S2

Supplementary Table S3

Supplementary Table S4

## Data Availability

The CUT&Tag-seq data that support the findings of this study have been deposited in the NCBI Gene Expression Omnibus (GEO; http://www.ncbi.nlm.nih.gov/geo/) with the accession number GSE310611. All the relevant data are available from the authors upon reasonable request.
