# Supplementary material for: Chronobiology of neurotropic viruses: rhythmic viral entry and arrhythmic host clocks
Source: Cell Discov. 2026 Feb 10;12:11. doi: 10.1038/s41421-026-00867-8 (PMC12886894; doi:10.1038/s41421-026-00867-8)

SUPPLEMENTARY INFORMATION

Supplementary Material and Methods

Cell culture conditions and reagents

The Cry1/2-double knockout MEF cells was a gift from Dr. Na Liu. The human glioblastoma cell line U-251 was a gift from Dr. Xiang Li. The mouse neuroblastoma cell line N2A was a gift from Professor Hao Yin. The human glioblastoma cell line SH-SY5Y, human embryonic kidney cells HEK293, and HEK293T were preserved in our laboratory. U-251, HEK293, and HEK293T cells were cultured at 37°C under 5% CO2 in Dulbecco's modified Eagle medium (DMEM; [HyClone, SH30022.01B]) supplemented with 10% fetal bovine serum (FBS; [Gibco, A5670701), 1% GlutaMAX™-I (Gibco, 35,050,061), 1% penicillin-streptomycin (P/S; [Gibco, 15,140,122]). N2A and SH-SY5Y cells were cultured at 37°C under 5% CO2 in Modified Eagle medium (MEM; [HyClone, SH30024.01]) supplemented with 10% FBS, 1% GlutaMAX™-I, 1% Non-Essential Amino Acids (NEAA; [Gibco, 11,140,035]).

Flag-tag affinity purification for mass spectrometry

HEK293 cells (1×10^7) were seeded into 15 cm dishes and incubated overnight, then transfection with Nishigahara-G-Flag and B19-G-Flag, respectively. At 60 h post-transfection, harvested and pelleted via centrifugation at 500 × g for 5min using a swinging bucket rotor. The cell pellet was washed once with cold PBS (Sangon Biotech, E607008), and then resuspended in 2 mL of cold IP buffer (containing 100 mM NaCl, 50 mM Tris-HCl, pH 7.4, 10 mM $MgCl_2$, 1% Nonidet p-40 [NP-40; Sigma-Aldrich, I3021], supplemented with protease inhibitor tablets [Sigma-Aldrich, S8820]). The cells were lysed on ice for 30 min at 4 °C and the lysates were subsequently clarified by centrifugation at 3,500 × g for 10min at 4°C. For input analysis, 60 µL aliquots of each lysate were mixed with 15 µL of 5× sample loading buffer (Beyotime Biotechnology, P0015L) to represent the whole cell lysate fraction. The remaining lysate was incubated with 25 µL of Anti-Flag-M2 agarose beads (Sigma-Aldrich, A2220) overnight at 4 °C with constant rotation. Following incubation, the beads were washed five times with 2 mL of IP buffer. Proteins were eluted and were resolved on SDS-PAGE gels to assess Strep-tagged protein expression by immunoblotting. The remaining eluate was used for mass spectrometric analysis.

Interferon β Measurement

Mouse brain tissue was collected after perfusion with PBS to remove blood. The brains were homogenized in PBS containing EDTA-Free Protease Inhibitor Cocktail (M5293, AbMole BioScience), followed by centrifugation at 4 °C for 10 min. The supernatant was used for ELISA analysis. The ELISA procedure was performed according to the instructions of the ELISA kit (SYP-M1594, purchased from UpingBio Technology Co., Ltd.; Hangzhou, China). Briefly, 50 µL of standards with different concentrations or samples were added to designated wells, followed by incubation with biotin-conjugated antibody at 37 °C for 60 min. Plates were washed five times with wash buffer, then incubated with HRP-conjugated streptavidin at 37 °C for 20 min. After washing, 100 µL of TMB substrate was added and incubated at 37 °C for 15 min in the dark. The reaction was stopped with 50 µL of stop solution, and absorbance was measured at 450 nm using a microplate reader. Sample concentrations were calculated from the standard curve using a four-parameter logistic regression model. IFN-β levels were normalized to total protein concentration determined by BCA assay and expressed as relative values.

CRISPR-Cas9 knockout and shRNA knockdown

The guide RNA (gRNA) was designed using SYNTHEGO's CRISPR design tool, and the shRNA sequences were designed using MERCK's shRNA design tool. Double-stranded oligonucleotides corresponding to the target sequences were cloned into lenti-CRISPR-V2 vector plasmids (for gene knockout) and PLKO.1 vector plasmids (for gene knockdown) to generate plasmids for gene knockout and knockdown in this study (detailed information in the supplementary table S4). These knockout plasmids or lenti-CRISPR-V2 (NC), as well as knockdown plasmids or PLKO.1 (NC), were transfected into 293T cells along with helper plasmids pSPAX and VSVG to produce lentiviruses. The lentiviruses were collected 48 h and 72 h after transfection, filtered through a 0.22 µm filter (Millipore, MA, USA),

and used to transduce glioblastoma cell lines. Single-cell clones were selected by puromycin and expanded in vitro. The results of knockout or knockdown were confirmed by immunoblotting after transduction with knockout lentiviruses as described above.

Western blots

For proteins with molecular mass below ~130 kDa, cells or thoroughly homogenized animal tissues were lysed in TAP lysis buffer for 30 min at 4 °C. Lysates were separated by SDS–PAGE using the Laemmli gel system and then transferred onto nitrocellulose membranes (Bio-Rad, 1620177). For high-molecular-weight proteins (HUWE1), cells were lysed in CHAPS lysis buffer for 30 min at 4 °C. Protein samples were resolved by LDS–PAGE and electrophoresed using a Tris–Acetate gel system (High Molecular Weight Protein Separation Gel Kit, Servicebio), then transferred onto nitrocellulose membranes with the stacking gel left intact. Membranes were blocked in 5% non-fat milk for 1 h at room temperature, then incubated with primary antibodies overnight at 4 °C. After three washes in PBS-T, membranes were incubated with HRP-conjugated secondary antibodies for 1 h at room temperature. Signal detection was carried out in a darkroom using an enhanced chemiluminescence (ECL) substrate (Thermo Fisher, 32106).

Real-time PCR

Total RNA was extracted according to the instructions of the RNAiso Plus kit (TAKARA, 9108). Subsequently, 1 μg of total RNA was reverse transcribed into cDNA using the MonScript cDNA™ RTII SuperMix with dsDNase (Monad Biotech Co., Ltd., MR05201). Fluorescence quantitative PCR reactions were conducted using a real-time fluorescence quantitative PCR instrument (Bio-Rad, CFX96). The comparative Ct method was employed to determine the relative mRNA expression of the target genes normalized to the housekeeping gene β-actin.

Dual-Luciferase Reporter Assay

HEK293 cells were co-transfected with reporter plasmids (p75NTR-Luc, BMAL1-Luc, BMAL1-dRORE) and indicated plasmids. Cells in 24-well plates were harvested and lysed 36 h after transfection, and luciferase activity was measured using the Dual-Glo Luciferase Assay System (Promega) according to the manufacturer's instructions. Luciferase activity was normalized to Renilla luciferase activity.

Supplementary tables

Table S1. List of Viruses and Their Receptors Hosted by Humans.

Please see Table S1.xlsx

Table S2. List of host proteins that bind to rabies virus strain Nishigahara glycoprotein identified by AP-MS.

Please see Table S2. xlsx

Table S3. List of host proteins that bind to rabies virus strain B19 glycoprotein Identified by AP-MS.

Please see Table S3. xlsx

Table S4. Oligonucleotide primers used during the course of this study.

Please see Table S4. xlsx

Supplementary figures

Supplementary Fig. S1. The expression of Neurotropic Viral Receptors is Regulated by the Circadian Clock

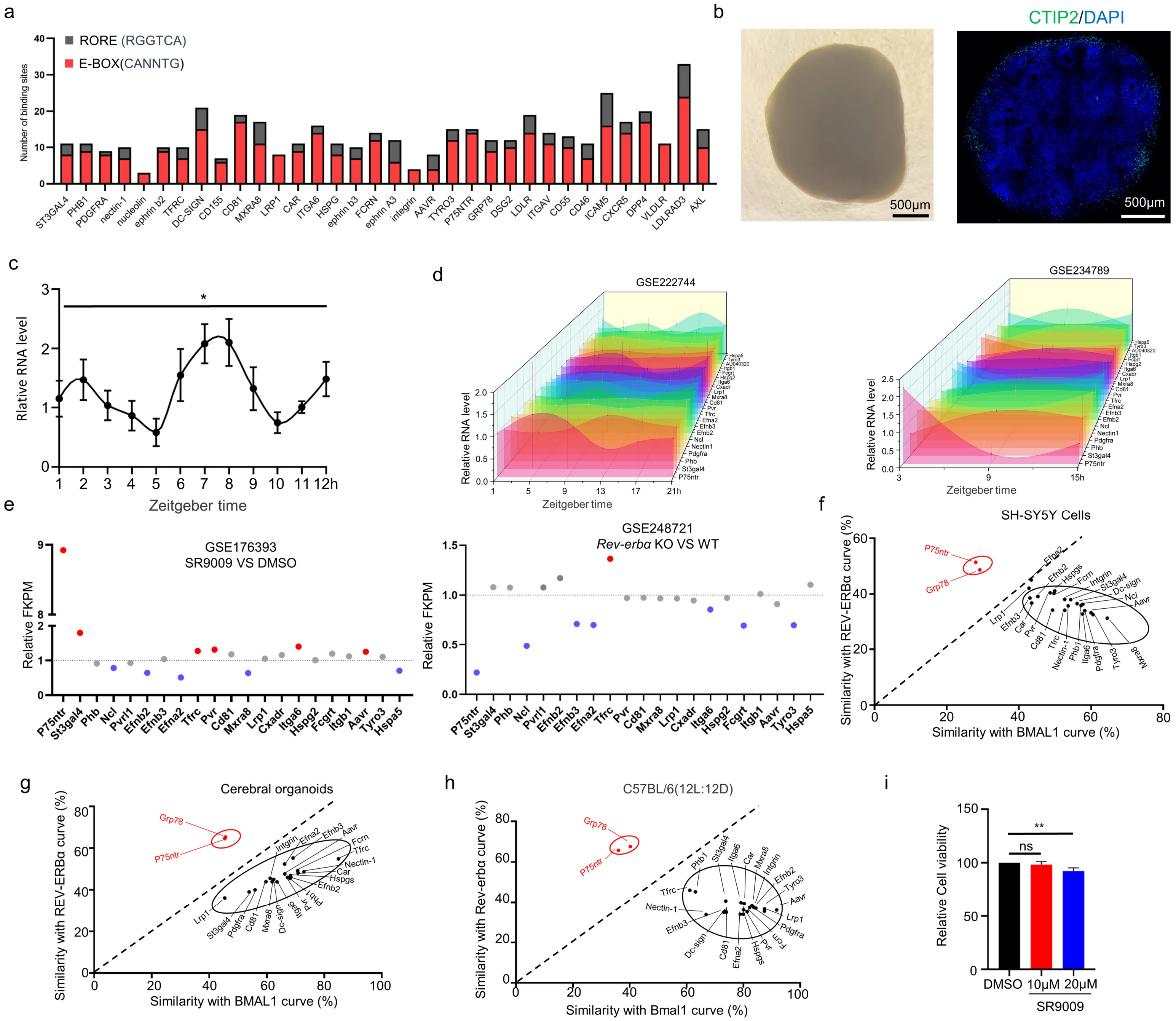

Supplementary Fig. S1. a, Analysis of transcription factor sites E-BOX (CANNTG) and RORE (RGGTCA) regulating the circadian clock on virus promoters. **b**, Bright-field imaging (left) and immunofluorescence images (right) of 60 DIV Cerebral organoids under the microscope. The detection of CTIP2 serves as a marker for characterizing neuronal differentiation and maturation in cerebral organoids. **c,** Cerebral organoids (60 DIV) were synchronized with 100 nM hydrocortisone, and total RNA was collected hourly from ZT0 to ZT12; *BMAL1* and *GAPDH* mRNA levels were quantified by qRT-PCR. **d, e**, Analysis of RNA-seq data from the dorsal hippocampus (GSE222744) and submandibular gland tissues (GSE234789) of mice (**d**). neurotropic viral receptors mRNA FKPM analysis on RNA-seq data from SR9009/shBmal1-treated Calu-3 cells (GSE176393) and U2OS WT/Rev-erbα KO lines (GSE248721) (**e**). **f-h**, Euclidean distance algorithm was utilized to assess the similarity of rhythmic expression patterns of various receptor genes in SH-SY5Y cells (**f**), cerebral organoids (**g**), and mouse brain (**h**), based on data from panels Fig. 1g-i, *BMAL1* and *REV-ERBα* served as reference gene, with the dashed line indicating equal similarity (y = x). **i**, Cell viability of SH-SY5Y cells following treatment with DMSO or SR9009 (10 μM or 20 μM) for 24 h, as determined by MTT assay. The data were presented as mean ± SEM. Statistical significance was determined using independent-sample t-tests, while rhythmicity was evaluated by CFJHC. *: *p*<0.05, **: *p*<0.01, ns: not significance.

Supplementary Fig. S2. Host Cell Susceptibility to Neurotropic Viruses is Regulated by Circadian Clock

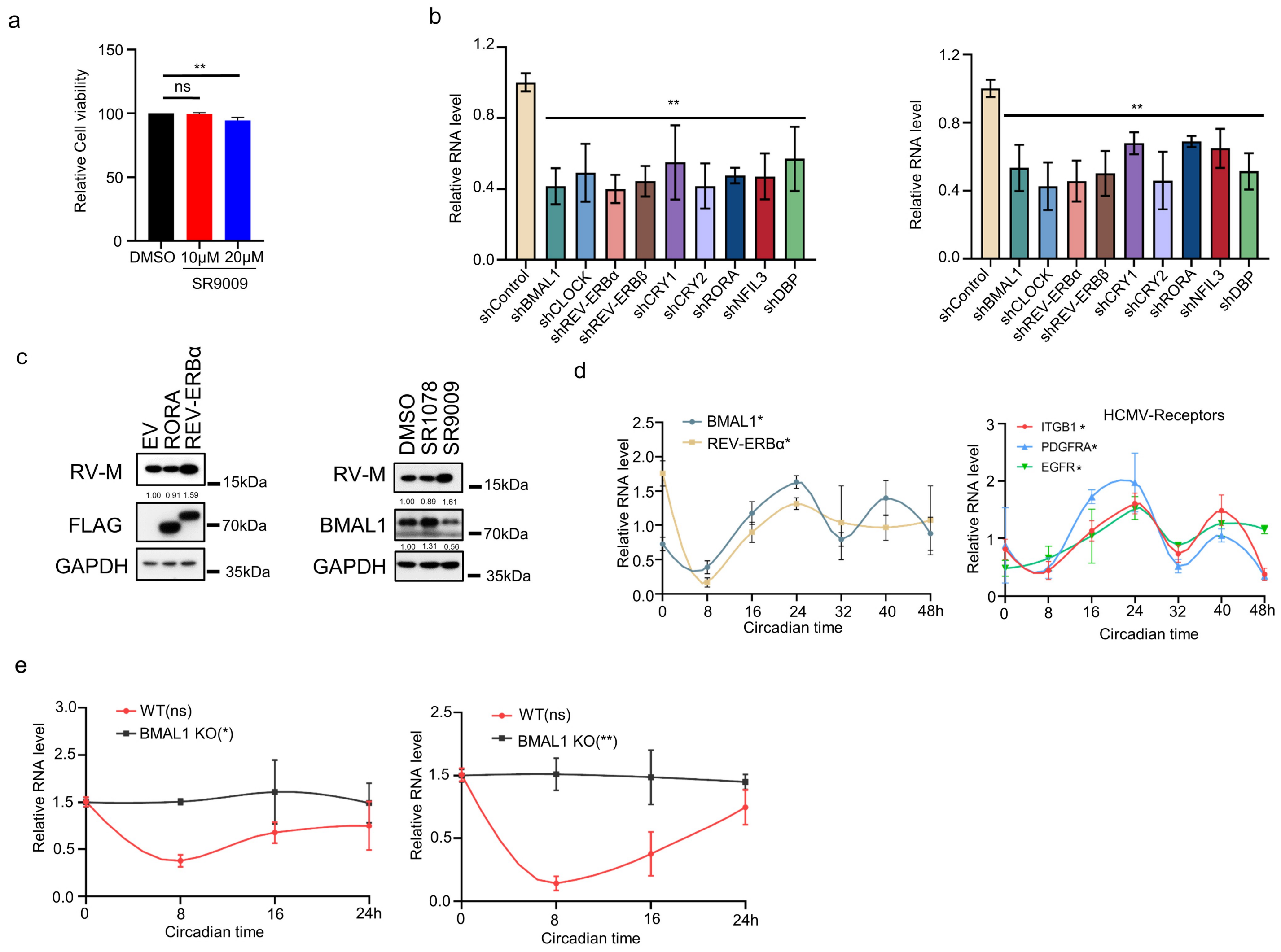

Supplementary Fig. S2. a, Cell viability of U-251 cells following treatment with DMSO or SR9009 (10 μM or 20 μM) for 24 h, as determined by MTT assay. **b,** qRT-PCR was performed to measure the mRNA levels of the clock gene of shRNA silenced SH-SY5Y (left) and U-251 (right) cells. **c,** SH-SY5Y cells were transfected to ectopically express EV, RORA, or REV-ERBα (left) or treated with DMSO, SR1078 (15 μM), or SR9009 (15 μM) for 24 h (right). Cells were subsequently infected with CVS-11 for 48 h, total protein was extracted, and Western blotting was performed to assess RABV-M, Flag-tagged protein levels (left) or BMAL1 (right), and GAPDH. **d,** Circadian mRNA expression of *BMAL1*, *REV-ERBα*, *ITGB1, PDGFRA* and *EGFR* in U251 cells post dexamethasone synchronization. Data normalized to the mean expression level. **e,** Synchronized WT or BMAL1-KO SH-SY5Y (left) and U251 (right) cells were lysed every 8 h, and *REV-ERBα* mRNA levels were assessed by qRT-PCR, normalized to CT0. Data are presented as mean ± SEM. The data are normalized to the mean value within each group, presented as mean ± SEM. Statistical significance was determined using independent-sample t-tests, while rhythmicity was evaluated by ARS or CFJHC. *: $p<0.05$, **: $p< 0.01$, ns: not significance.

Supplementary Fig. S3. E2F8 Repression by REV-ERBα Augments p75NTR Expression and RABV Entry

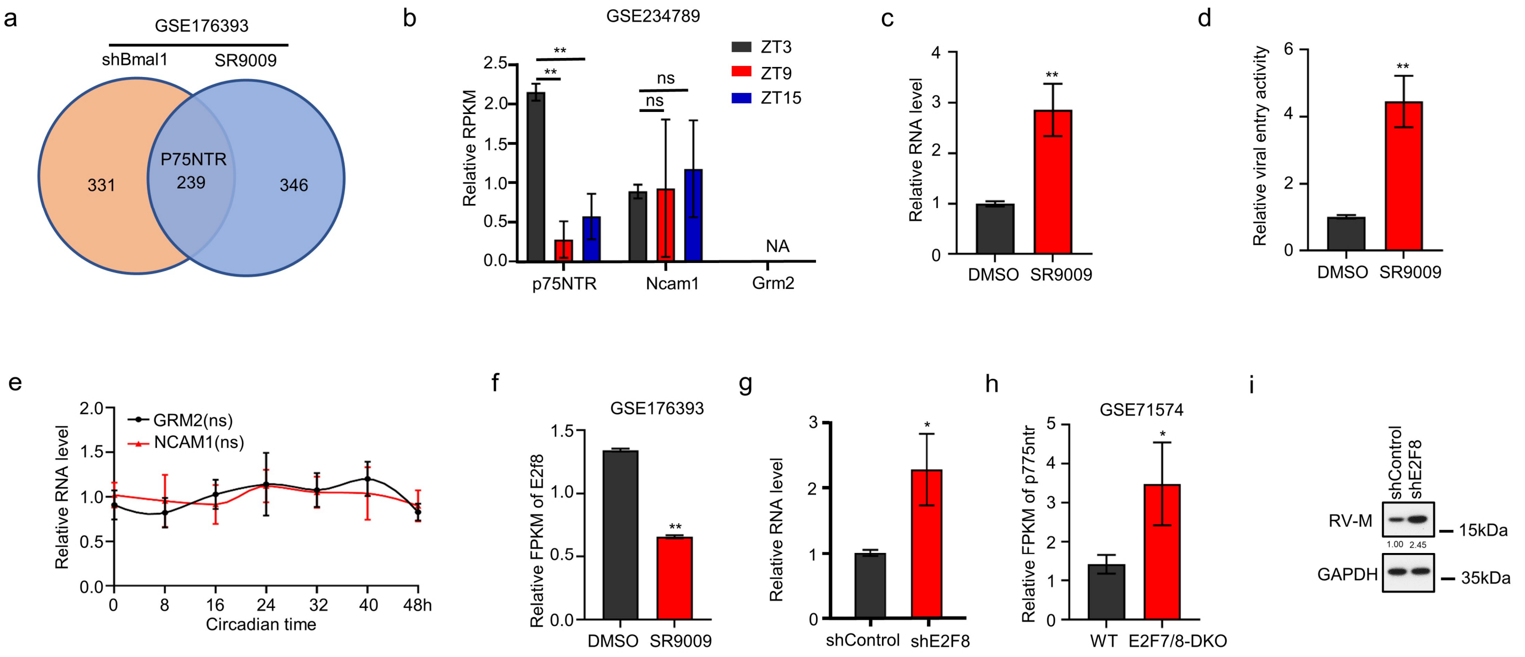

Supplementary Fig. S3. a, b, Analysis of RNA-seq data from SR9009- or shBmal1-treated Calu-3 cells (GSE176393) (**a**) and submandibular gland tissues of mice (GSE234789) (**b**). **c,** qRT-PCR measurement of *p75NTR*and *GAPDH* mRNAs levels in DMSO- or SR9009-treated SH-SY5Y cells. **d,** SH-SY5Y cells were treated with DMSO or SR9009 for 24 h, followed by infection with CVS-11 (MOI = 5) for 0.5 h. Viral genome and *GAPDH* mRNA levels were measured by qRT-PCR. **e,** Total RNA and protein lysates were extracted from SH-SY5Y cells synchronized with a 100 nM dexamethasone pulse. mRNA levels of *GRM2, NCAM1* and *GAPDH* were detected by qRT-PCR. **f,** The RNA-seq data of Calu-3 cells treated with DMSO or SR9009 in GSE176393 dataset were selected for analysis *E2F8* FPKM. **g,** Total RNA was extracted from E2f8-silenced SH-SY5Y cells, the mRNA levels of *p75NTR* and *GAPDH* were measured by qRT-PCR. **h,** GSE71574 dataset analysis showed that E2f7/8 DKO can up-regulate the mRNA level of p75NTR. The RNA-seq data of normal mouse liver tissues (WT and E2f7/8DKO) in GSE71574 dataset were selected for analysis. **i,** Post CVS11 infection 48 h in control/E2F8-silenced SH-SY5Y cells, RABV-M protein and *GAPDH* were analyzed by Western blot. The data are normalized to the mean value within each group, presented as mean ± SEM. Statistical significance was determined using independent-sample t-tests, while rhythmicity was evaluated by ARS. *: *p*<0.05, **: *p*< 0.01, ns: not significance.

Supplementary Fig. S4. E2F8 Reciprocal Feedback with REV-ERBα Modulates Host-Virus Interactions

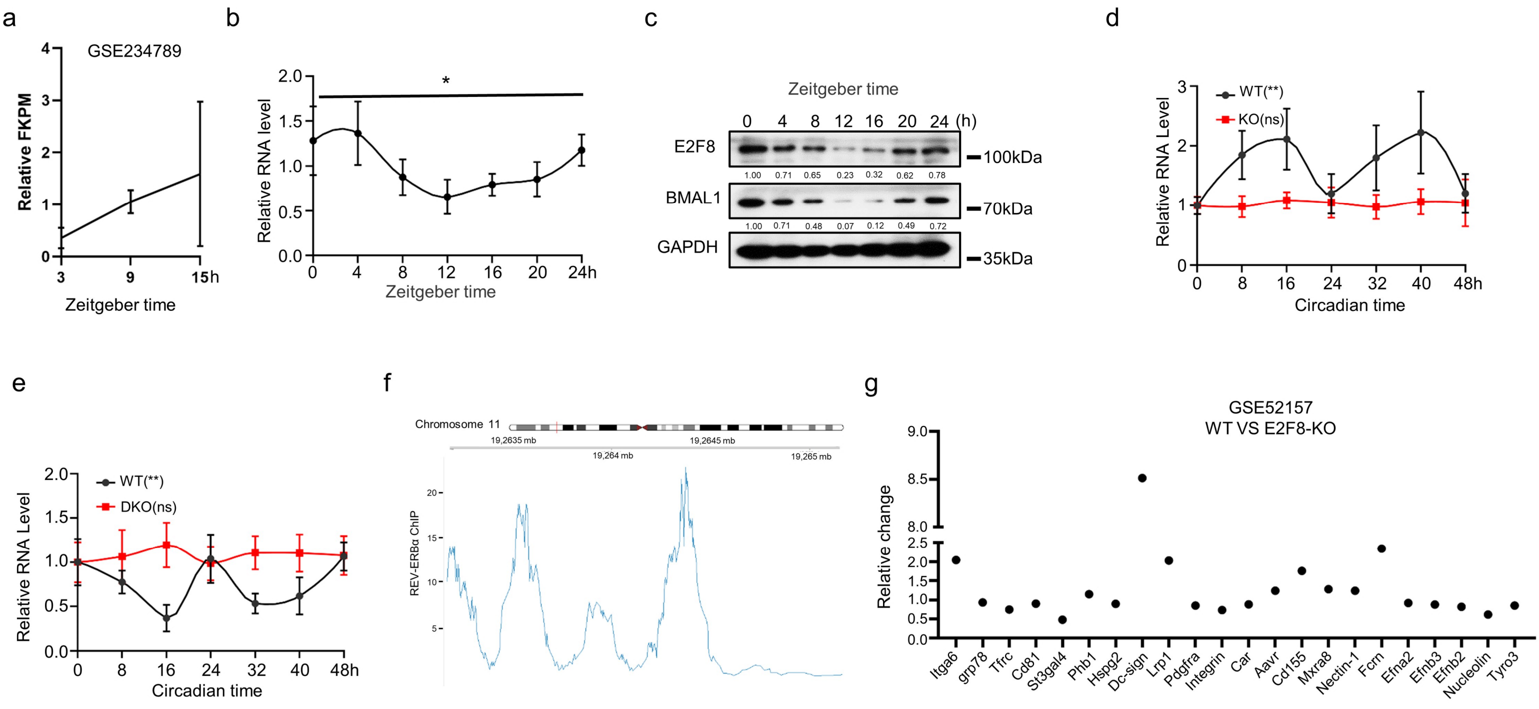

Supplementary Fig. S4. a, Analysis of the GSE234789 dataset showing rhythmic changes in *E2f8* mRNA levels in mouse submandibular gland tissues. **b, c,** Total RNA and protein lysates were extracted from mouse brain tissues at the indicated Zeitgeber times. mRNA levels of *E2f8* and *GAPDH* were detected by qRT-PCR (**b**), and protein levels of E2f8, Bmal1, and GAPDH were assessed by western blotting (**c**). **d, e,** WT/BMAL1-KO SHSY5Y cells (**d**) or WT/Cry1/2-DKO MEF cells (**e**) were synchronized with 100 nM dexamethasone and sampled at 8 h intervals from CT0 to CT48. *E2F8* mRNA levels were quantified by qRT-PCR and normalized to *GAPDH*. **f,** The bigWig file from the GSM8123058 dataset was processed using the rtracklayer and GenomicRanges packages in R (version 4.4.2). Results were visualized with ggplot2 for clarity. **g,** Analysis of RNA-seq data from spleen Ter119^{+} CD71high cells of WT and E2F8 KO mice (GEO dataset GSE52157). The data are normalized to the mean value within each group, presented as mean ± SEM. Statistical significance of rhythmicity was evaluated by ARS or CFJHC. *: *p*<0.05, **: *p*< 0.01, ns: not significance.

Supplementary Fig. S5. Comparison of food intake between mock-infected and RABV-infected mice

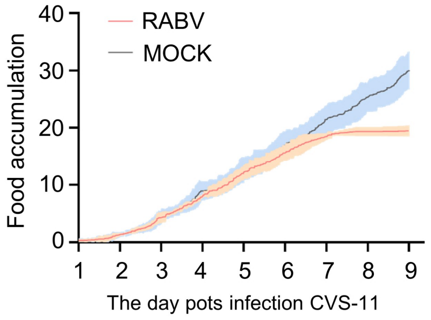

Supplementary Fig. S5. Male mice (12 weeks old) were divided into RABV-infected (CVS-11 strain) and mock-infected (PBS) groups. Food intake were monitored post-infection using the Lab Animal Monitoring System.

Supplementary Fig. S6. The glycoproteins of VSV and EBOV do not alter the half-life of REV-ERBα or its degradation induced by Li^+

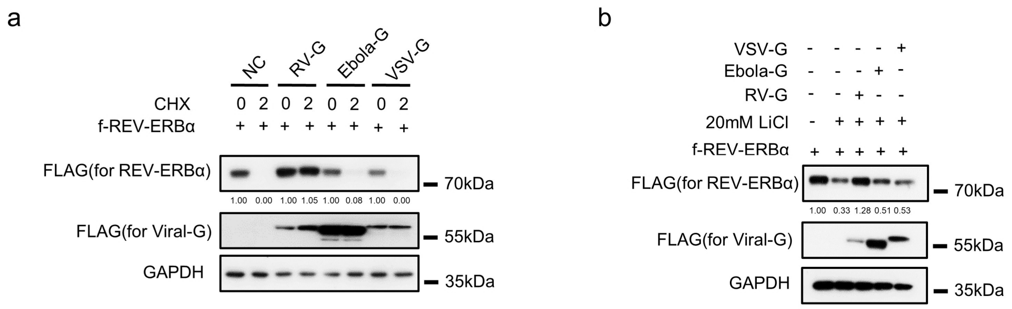

Supplementary Fig. S6. a, Immunoblot analysis of extracts from HEK293 cells transfected with REV-ERBα-FLAG and treated with MG132 (100 μM) or overexpressing RV-G/VSV-G/EBOV-G for 24 h, followed by treatment with cycloheximide (CHX; 25 μg/mL) for 0 h or 2 h. **b**, Western blot of WT-REV-ERBα-Flag protein level in HEK293 cells which transfected with EV/RV-G/ VSV-G/EBOV-G, then treated with 20 mM LiCl for 12 h.

Supplementary Fig. S7. Rhythmic Variations in REV-ERBα, BMAL1 and RABV receptors mRNA Levels in the Mouse Brain Under Regular Light-Dark Cycles and Constant Darkness

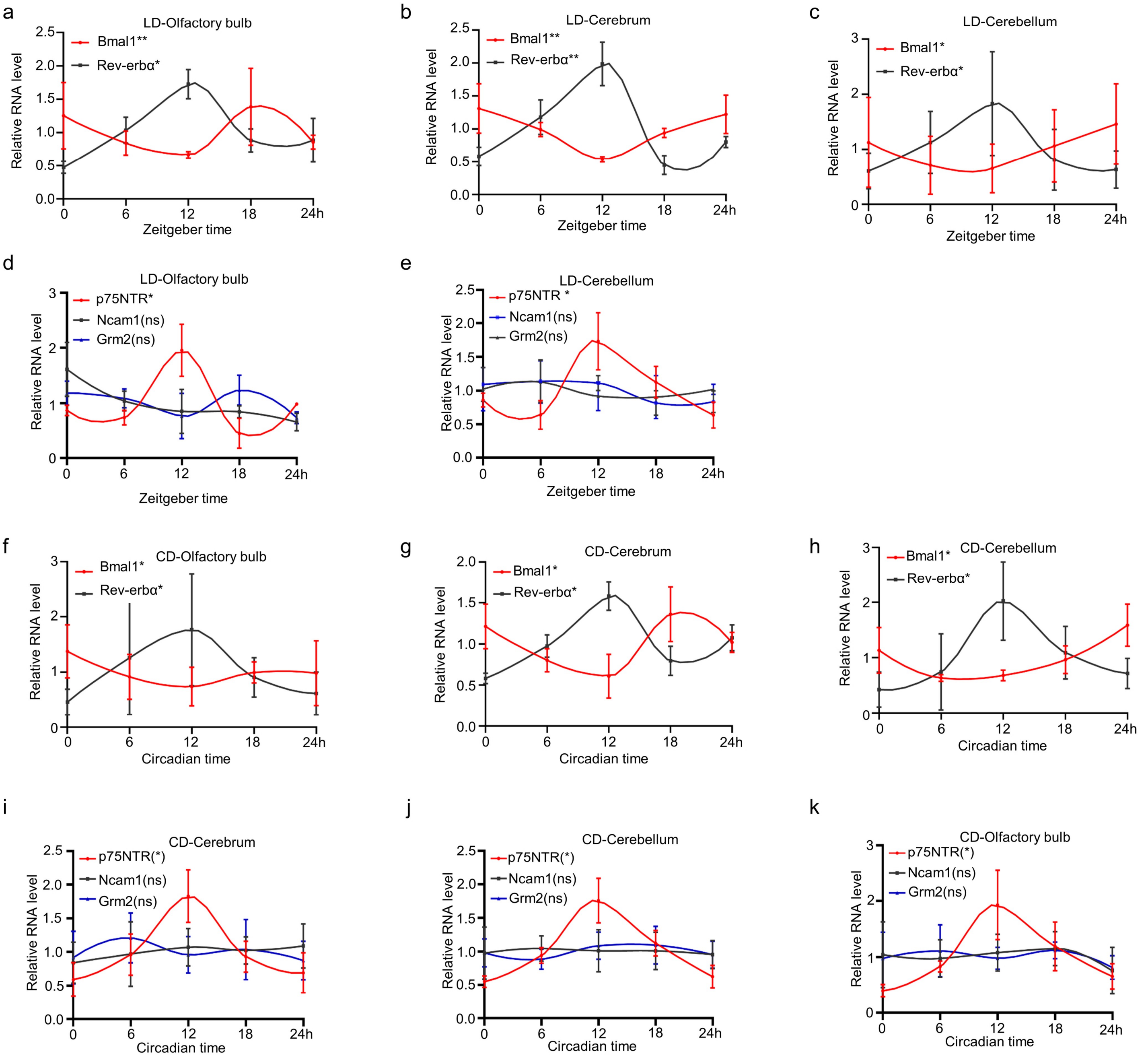

Supplementary Fig. S7. a-e Male wild-type C57BL/6 mice were housed under a 12:12 light-dark cycle (lights on: 8 am, lights off: 8 pm) for three weeks. Starting at 8:00 am (ZT0), mice were euthanized every 6 h, and brain tissues were collected to measure mRNA levels of BMAL1, REV-ERBα, and GAPDH in the olfactory bulb (**a**), cerebellum (**b**), and cerebrum (**c**) via qRT-PCR. Additionally, mRNA levels of RABV receptors were measured in the olfactory bulb (**d**) and cerebellum (**e**) (*n* = 5). **f-k** After three weeks under regular light conditions, C57BL/6 mice were maintained in constant darkness for three days before sampling at circadian times (CT) 0, 6, 12, 18, and 24. qRT-PCR was used to assess mRNA levels of BMAL1, REV-ERBα, and GAPDH in the olfactory bulb (**f**), cerebellum (**g**), and cerebrum (**h**), and RABV receptor mRNA levels in the olfactory bulb (**i**), cerebellum (**j**), and cerebrum (**k**) (*n* = 5). LD: Light/dark; CD: constant darkness. The data are normalized to the mean value within each group, presented as mean ± SEM. Statistical significance of rhythmicity was evaluated by ARS or CFJHC. *: $p<0.05$, **: $p< 0.01$, ns: not significance.

Supplementary Fig. S8. CJL Does Not Alter mRNA Levels of p75NTR but Affects the Rhythmic Pattern of Clock Gene Transcripts BMAL1 and REV-ERBα

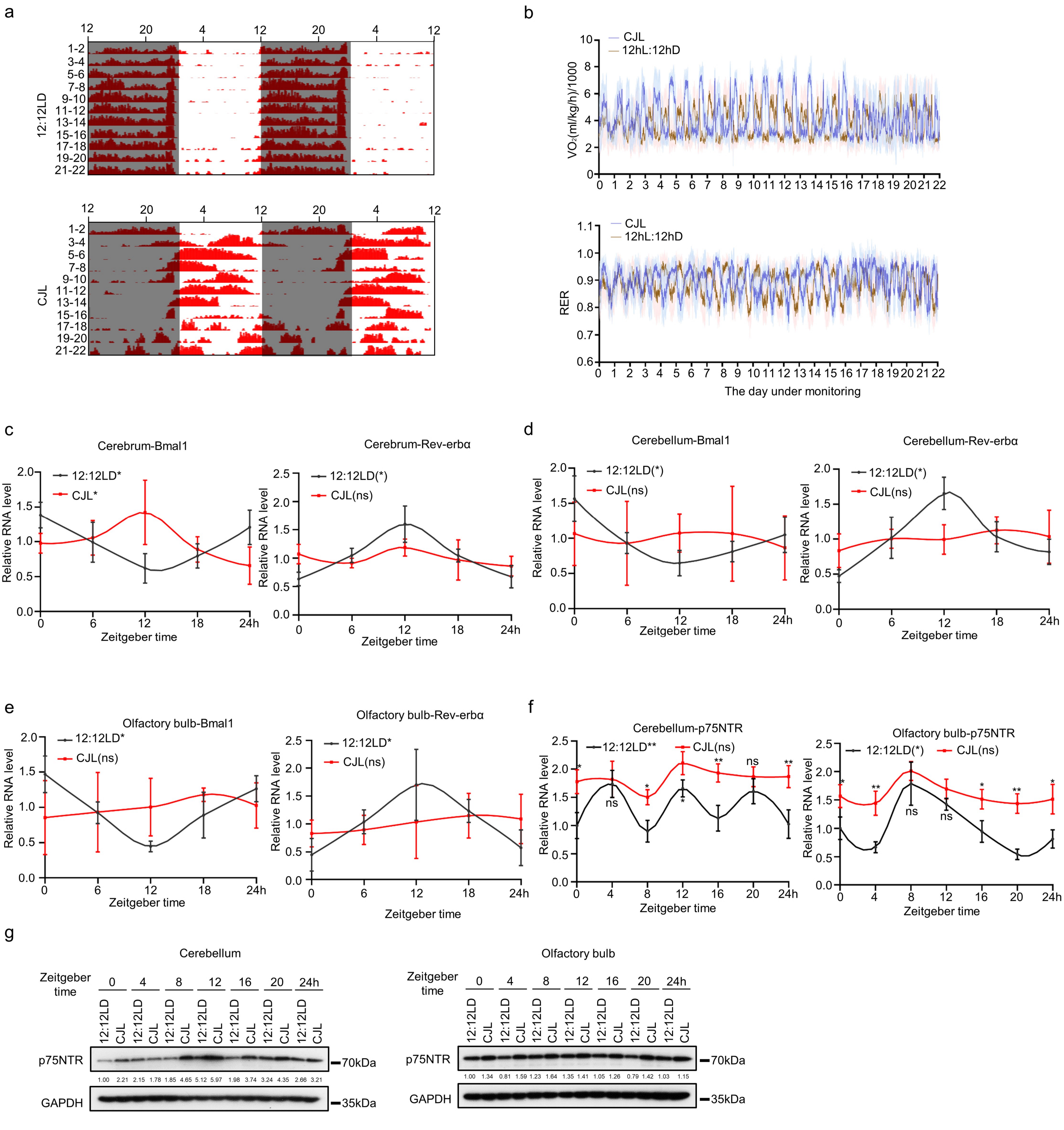

Supplementary Fig. S8. a, b, Running wheel activity (**a**), oxygen consumption and RER (**b**) of mice under regular LD or CJL conditions were monitored via the Comprehensive Lab Animal Monitoring System. **c-e** Brain tissues from CJL and 12:12 LD mice were collected at Zeitgeber times 0, 6, 12, 18, and 24. qRT-PCR was used to measure mRNA levels of clock genes *Bmal1*, *Rev-erbα*, and *GAPDH* in the cerebrum (**c**), cerebellum (**d**), and olfactory bulb (**e**). **f, g,** Olfactory bulb and cerebellar tissues were collected from CJL mice or 12:12 L/D mice at different zeitgeber times. p75NTR mRNA levels were measured by qRT-PCR and normalized to the mean p75NTR mRNA level at ZT0 in 12:12 L/D mice (**f**). Data are presented as mean ± SEM (*n* = 5). Protein levels of p75NTR and GAPDH in the olfactory bulb and cerebellum of CJL and 12:12 L/D mice at different zeitgeber times were assessed by Western blot (**g**). Panels c-e were normalized to each group mean, while panel f was normalized to the control mean at ZT0, data are shown as mean ± SEM. The significance of rhythmicity was evaluated by ARS or CFJHC. *: $p<0.05$, **: $p< 0.01$, ns: not significance.

Supplementary Fig. S9. REV-ERBα knockout does not affect rhythmic behavior and rhythmic physiological functions in mice

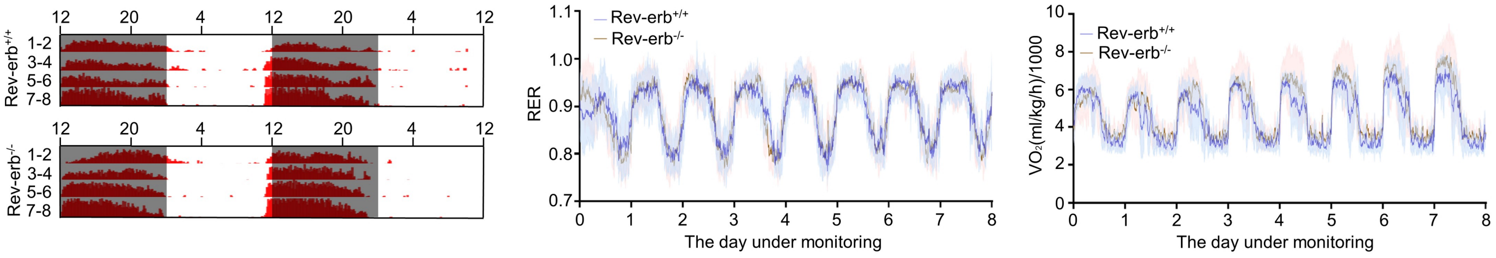

Supplementary Fig. S9. Running wheel, oxygen consumption and RER of WT or REV-ERBα$^{-/-}$ mice were monitored via the Comprehensive Lab Animal Monitoring System.

Supplementary Fig. S10. Enhanced the clinical signs, viral load and the expression of inflammatory genes in mice infected with RABV at ZT12 compared to ZT0.

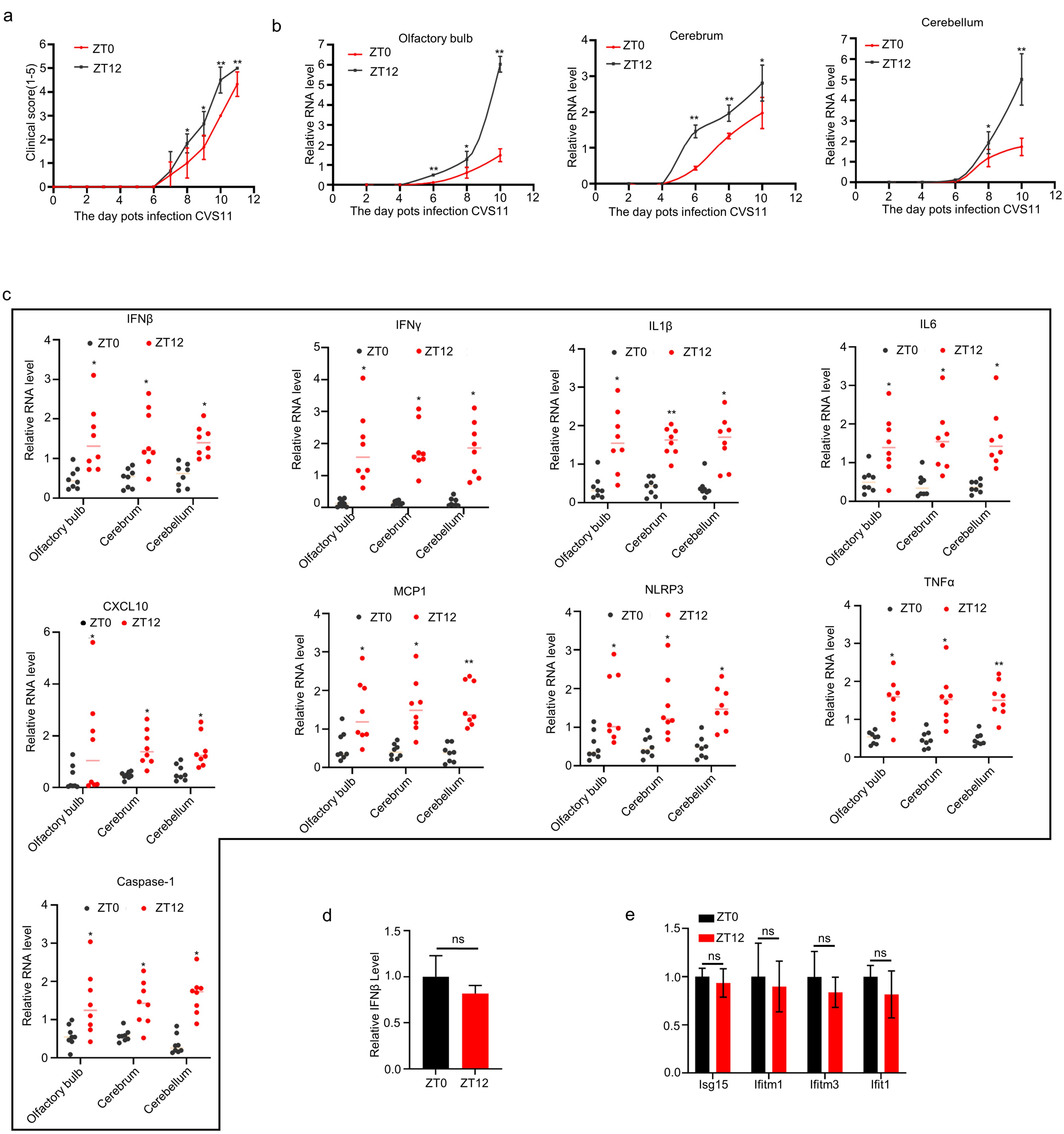

Supplementary Fig. S10. a, Clinical signs in mice infected with CVS-11 at ZT0 or ZT12. Data are presented as mean ± SEM (*n* = 8). **b,** Following CVS-11 infection, brain tissues were collected every two days. Rabies virus genome and *GAPDH* mRNA levels in various brain regions were quantified by qRT-PCR. Data are expressed relative to the mean (*n* = 5). **c,** Mice were infected with CVS-11 at ZT0 (*n* = 8) or ZT12 (*n* = 8). On the tenth day post-infection, RNA was extracted from the olfactory bulb, cerebrum, and cerebellum of euthanized mice. mRNA levels of inflammatory genes were measured by qRT-PCR. **d, e,** Brain tissues were collected from 12:12L/D mice at the ZT0 and ZT12. IFNβ secretion was measured by ELISA and normalized to total protein concentration to obtain relative IFNβ secretion (**d**). ISGs mRNA levels were determined by qRT-PCR (**e**). Data were normalized to the control mean and presented as mean ± SEM (*n* = 6). Significance was tested using independent-samples T test, *: $p<0.05$, **: $p< 0.01$, ns: not significance.

Supplementary Fig. S11. Elevated the clinical signs and the expression of inflammatory genes in CJL mice compared to 12:12LD mice

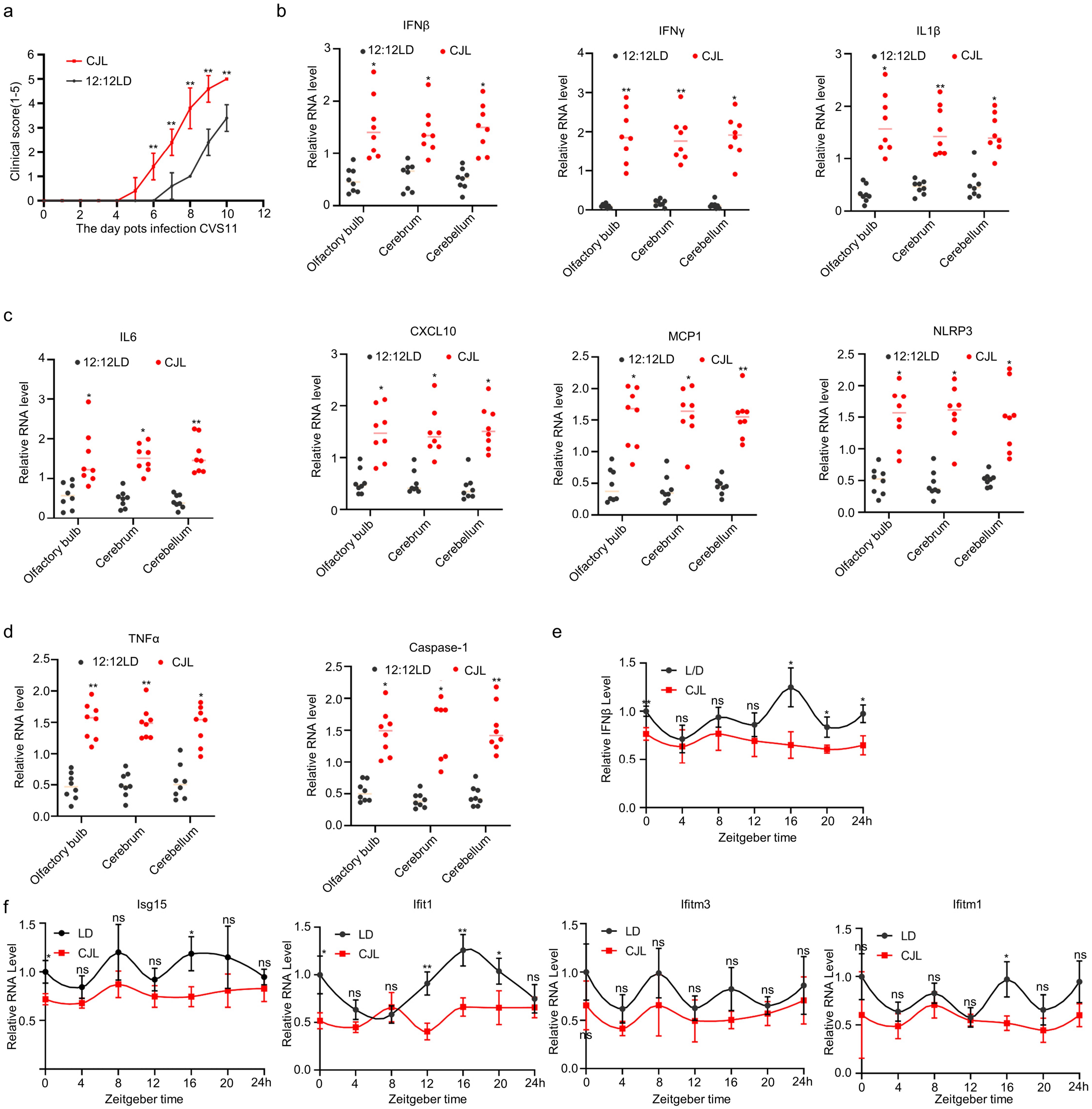

Supplementary Fig. S11. a, Clinical signs in CVS-11-infected mice under 12:12 LD or CJL conditions. Data are presented as mean ± SEM (*n* = 8). **b-d,** On the ninth-day post-infection with CVS-11, RNA was extracted from the olfactory bulb, cerebrum, and cerebellum of euthanized mice. mRNA levels of inflammatory genes were measured by qRT-PCR. **e,** f, Brain tissues were collected from CJL mice/12:12L/D mice at the indicated zeitgeber times. IFNβ secretion was measured by ELISA and normalized to total protein concentration to obtain relative IFNβ secretion (**e**). ISGs mRNA levels were determined by qRT-PCR (**f**), those data were normalized to the mean relative IFNβ secretion or ISG mRNA level at ZT0 in 12:12 L/D mice. Data are presented as mean ± SEM (*n* = 5). Significance was tested using independent-samples T test, *: *p*<0.05, **: *p*< 0.01, ns: not significance.

Supplementary Fig. S12. Decreased the clinical signs and the expression of inflammatory genes in the brain of infected Rev-erbα$^{-/-}$ mice

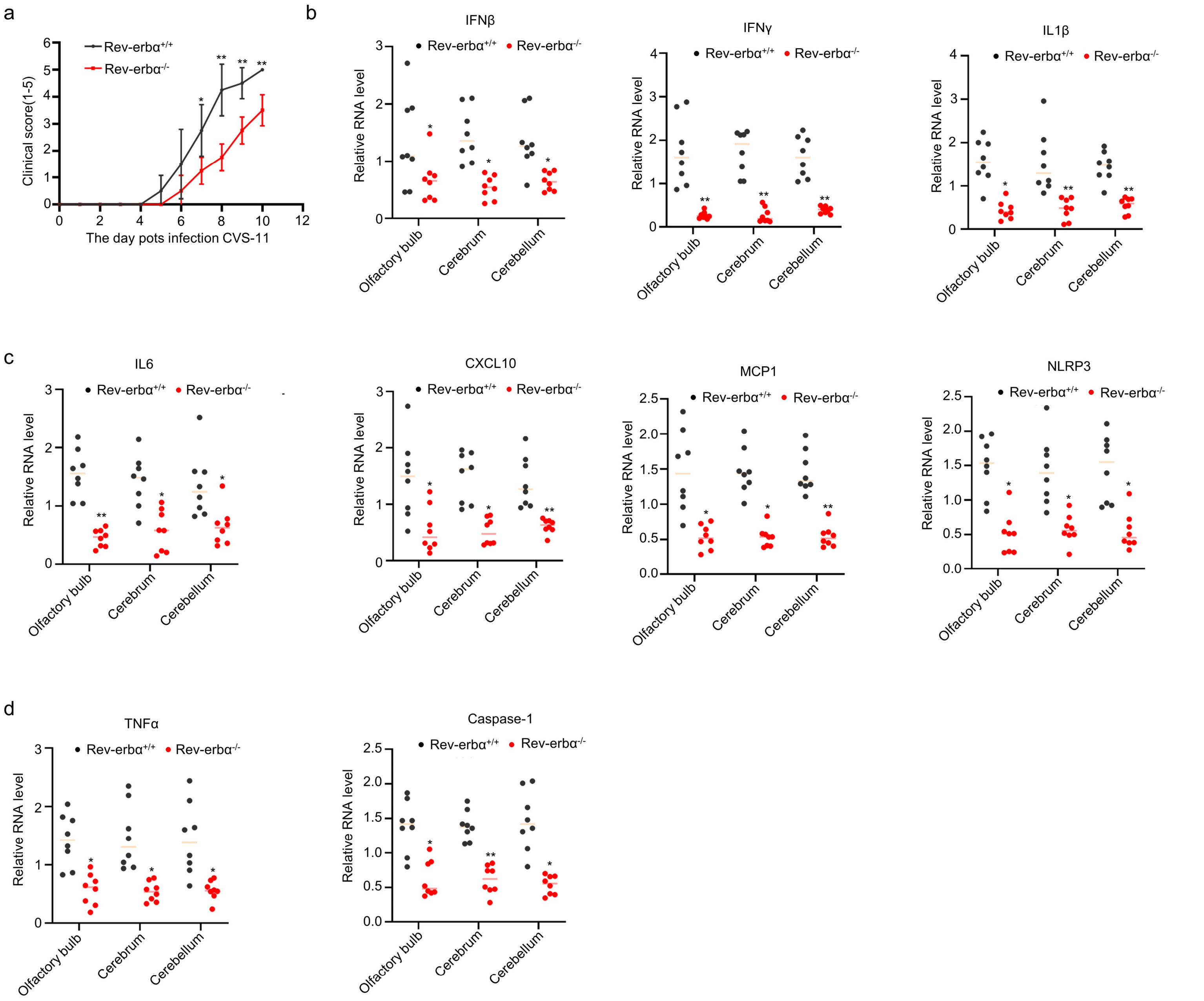

Supplementary Fig. S12. a, Clinical signs in wild-type and REV-ERBα$^{-/-}$ mice infected with CVS-11 (n = 8). **b-d,** Total RNA was extracted from the olfactory bulb, cerebrum, and cerebellum of 8-week-old male wild-type and REV-ERBα$^{-/-}$ mice on the ninth-day post-infection with CVS-11. mRNA levels of inflammatory genes were measured by qRT-PCR. Data are expressed relative to the mean, and statistical significance was determined using an independent-samples t-test, *: p<0.05, **: p< 0.01.

Supplementary Fig. 13. Graphic abstract

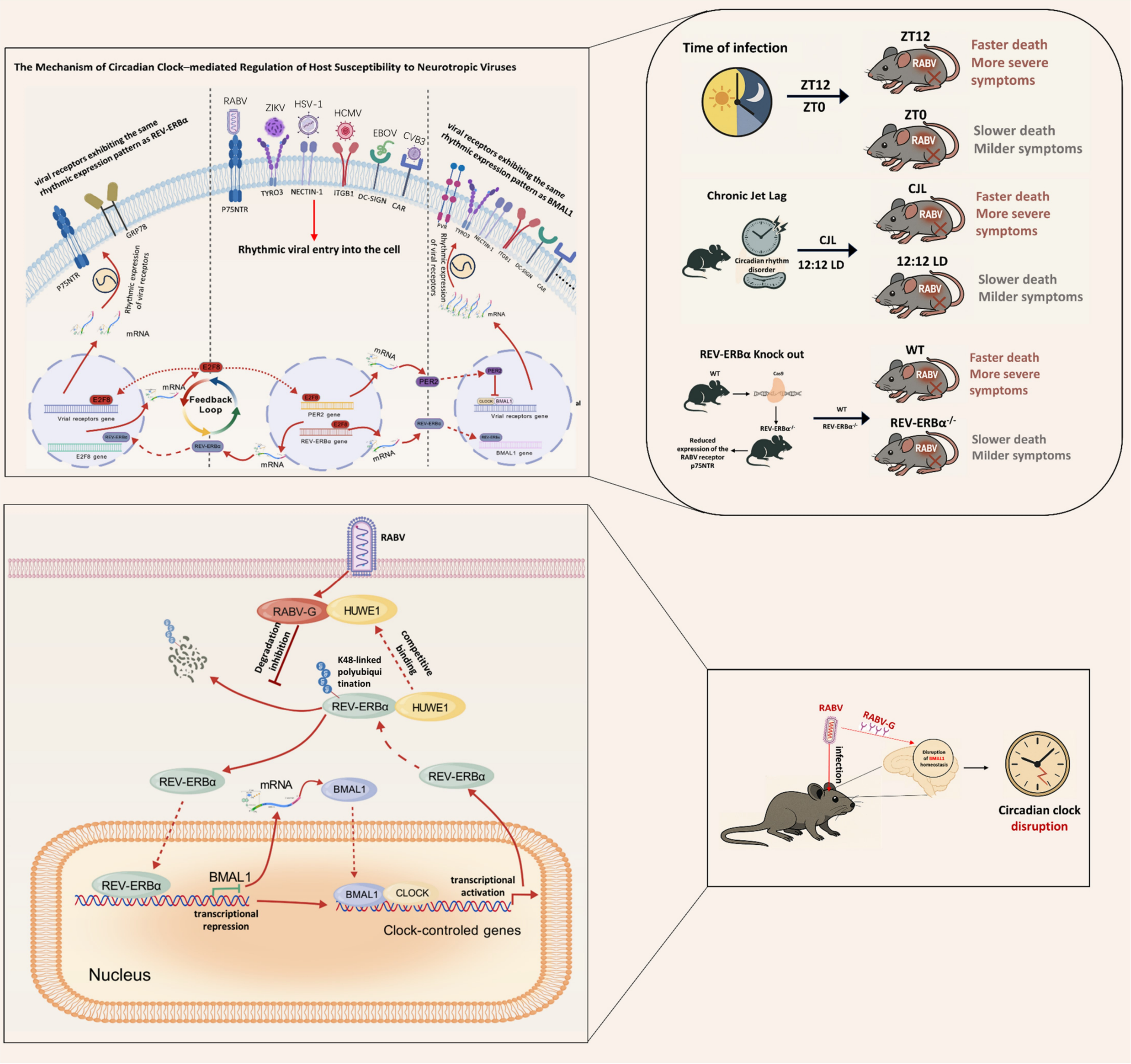

Supplement: Supplementary file 1 — Supplementary Information [file 41421_2026_867_MOESM1_ESM.pdf]
